# Plasmon-Induced Hot Electrons in Nanostructured Materials: Generation, Collection, and Application to Photochemistry

**DOI:** 10.1021/acs.chemrev.4c00165

**Published:** 2024-06-03

**Authors:** Li Zhou, Qijia Huang, Younan Xia

**Affiliations:** † The Wallace H. Coulter Department of Biomedical Engineering, Georgia Institute of Technology and Emory University, Atlanta, Georgia 30332, United States; ‡ School of Physics and Technology, Wuhan University, Wuhan, Hubei 430072, P. R. China; § School of Chemistry and Biochemistry, Georgia Institute of Technology, Atlanta, Georgia 30332, United States

## Abstract

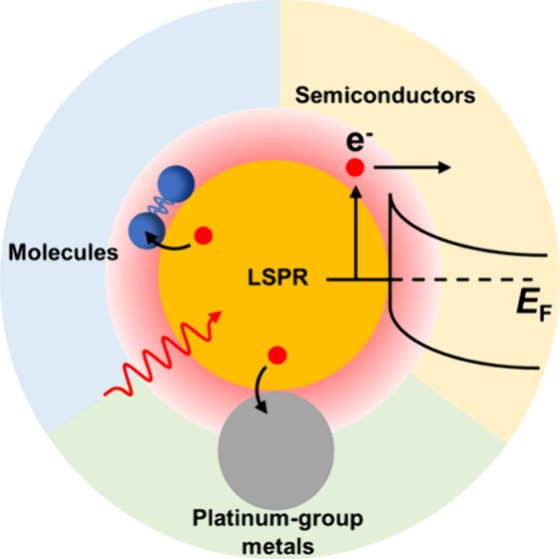

Plasmon refers to the coherent oscillation of all conduction-band electrons in a nanostructure made of a metal or a heavily doped semiconductor. Upon excitation, the plasmon can decay through different channels, including nonradiative Landau damping for the generation of plasmon-induced energetic carriers, the so-called hot electrons and holes. The energetic carriers can be collected by transferring to a functional material situated next to the plasmonic component in a hybrid configuration to facilitate a range of photochemical processes for energy or chemical conversion. This article centers on the recent advancement in generating and utilizing plasmon-induced hot electrons in a rich variety of hybrid nanostructures. After a brief introduction to the fundamentals of hot-electron generation and decay in plasmonic nanocrystals, we extensively discuss how to collect the hot electrons with various types of functional materials. With a focus on plasmonic nanocrystals made of metals, we also briefly examine those based upon heavily doped semiconductors. Finally, we illustrate how site-selected growth can be leveraged for the rational fabrication of different types of hybrid nanostructures, with an emphasis on the parameters that can be experimentally controlled to tailor the properties for various applications.

## 1 Introduction

**Figure 1:**
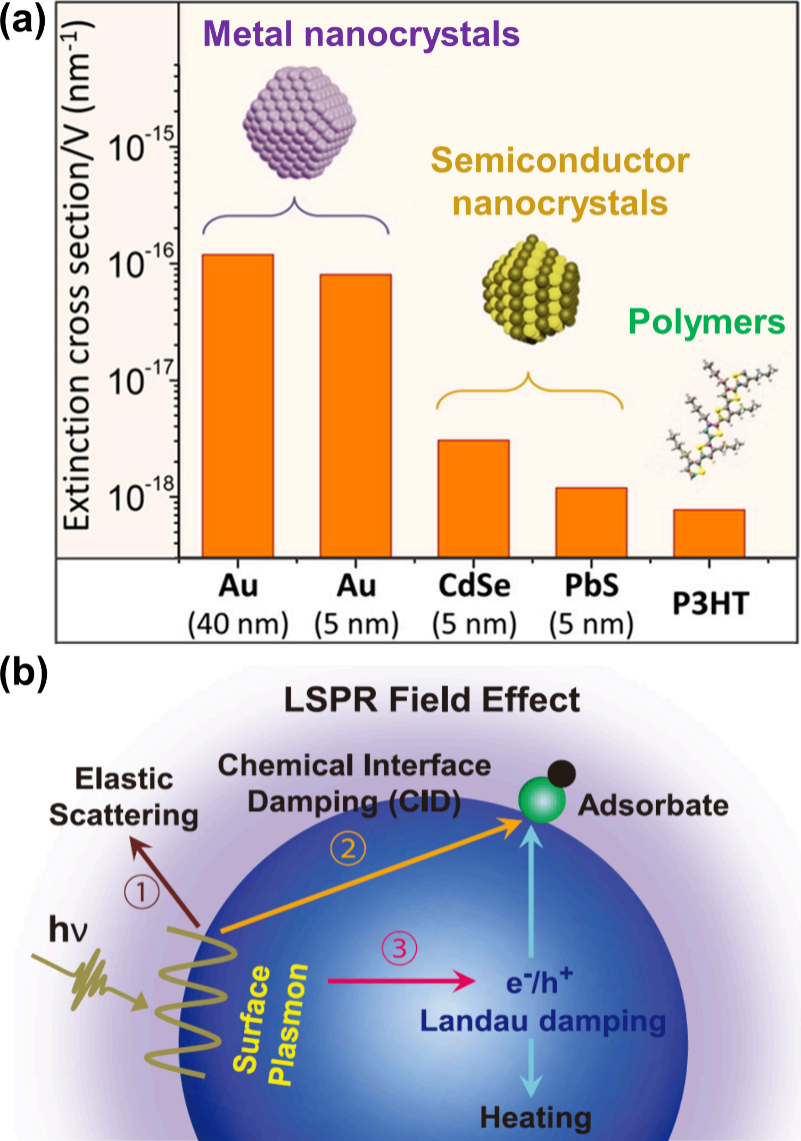
(a) Comparison of the extinction (absorption plus scattering) cross sections of some light-harvesting transducers, as exemplified by metal and semiconductor nanocrystals, as well as π-conjugate polymers. For the metal and semiconductor nanocrystals of 5 nm in size, extinction is dominated by absorption. Adapted with permission from ref (16). Copyright 2014 American Chemical Society. (b) Optical excitation and decay of LSPR with a large absorption cross-section and strong local field enhancement. The LSPR can decay through different channels: (1) radiative decay in the form of elastic scattering; (2) nonradiative resonant energy transfer, including chemical interface damping (CID) to a surface adsorbate or plasmon resonance energy transfer (PRET) to an adjacent semiconductor nanocrystal; and (3) nonradiative Landau damping accompanied by the generation of hot carriers and heating. Reproduced with permission from ref (12). Copyright 2016 IOP Publishing.

Understanding and manipulating light-matter interactions are essential to a variety of applications, including those related to photovoltaics,^1,2^ photodetection,^3,4^ photochemistry (e.g., photocatalysis and photoelectrochemical energy conversion),^5,6^ and photon-enabled diagnostics and/or therapy.^7,8^ In addition to scattering, absorption is another type of basic interaction, by which light is absorbed and converted to the internal energy of the matter. Metal/semiconductor nanocrystals, organic compounds, and polymeric materials have all been explored as the antennas (or transducers) to absorb light over a broad spectrum of wavelengths extending from ultraviolet (UV) to visible and near-infrared (NIR) regions. Among them, metal nanocrystals capable of supporting localized surface plasmon resonance (LSPR) have attracted the most attention in recent years owing to their extraordinarily large absorption cross sections.^9^ As illustrated in Figure 1a, the absorption cross sections of metal-based plasmonic nanocrystals are typically several orders of magnitude greater than those of semiconductor nanocrystals and organic molecules/materials, making them superb antennas for light absorption and related applications.^10−14^

LSPR refers to the coherent oscillation of all the conduction-band electrons in a plasmonic nanostructure made of a metal or a heavily doped semiconductor. The metal nanocrystals equipped with LSPRs exhibit superior light-harvesting efficiency, possessing an absorption cross-section that is 1–2 orders of magnitude larger than that of semiconductor nanocrystals and organic dyes (Figure 1a).^15−17^ The strong confinement and enhancement of local electric fields intrinsic to LSPR can be utilized to augment a variety of optical processes such as scattering, absorption, emission, and energy transfer.^18^ In addition, the spectral response of LSPR is highly tunable, depending on the elemental composition, size, and shape of the plasmonic nanostructure. Upon excitation, the plasmon can decay through different channels, offering a range of capabilities to convert the incident light to different forms of energy (Figure 1b). The decay of a plasmon through the radiative channel leads to intense optical scattering, which can be utilized to generate a light-trapping effect and thus enhance the absorption of light by an adjacent component situated in a heteronanostructure. In parallel, the plasmon can decay through a nonradiative channel for the initiation of a number of processes involving electrons, photons, and phonons.^19^ Particularly, the plasmon-induced energetic carriers, known as hot electrons and holes, arising from the dephasing of a plasmon and then nonradiative Landau damping have been leveraged to improve the performance of many types of light-driven devices, including solar cells, photodetectors, and photocatalytic reactors.

The nonradiative decay used to be viewed as a plasmon loss and thus considered a major obstacle to an array of applications involving amplification and propagation of plasmons.^20^ Major efforts have been devoted to overcoming this disadvantage by prolonging the lifetime of a plasmon and/or extending its propagation length. Specifically, metal–semiconductor and metal–dye hybrid nanostructures have been designed to mitigate plasmon loss through energy transfer from the semiconductor or organic dye to the plasmonic unit. In this way, plasmon amplification has been realized.^21,22^ Alternatively, by leveraging plasmonic nanostructures as light-harvesting antennas, nonradiative decay offers a powerful means to convert the absorbed photons to various forms of energy in high efficiency, including the creation of hot carriers. To this end, plasmonic nanocrystals have been integrated with different types of functional molecules or materials (including catalytic metals and semiconductors) for the fabrication of heteronanostructured systems. Research themes involving theoretical inquiry,^15^ dynamic analysis,^19,23^ and detection^24^ of plasmon-induced hot carriers have drawn widespread attention. Benefiting from the large absorption cross sections of metal nanocrystals, the plasmon-induced hot carriers hold great promises for photodetective,^4^ photovoltaic,^5^ and photochemical applications.^12,13,25^ All these applications call for the rational design and controlled fabrication of plasmon-based hybrid nanostructures.

This review focuses on the generation and transfer of plasmon-induced hot electrons in a variety of hybrid nanostructures. Although both hot electrons and holes can be utilized, most of the current studies have concentrated on hot electrons because of their higher mobility and faster transfer kinetics. That is why we place the focus of this review article on hot electrons. We start with a brief introduction to the fundamentals of hot-electron generation and decay in plasmonic nanocrystals, followed by extensive discussion on how to collect the hot electrons with various types of functional materials for an array of applications. While our discussions focus on plasmonic metals, we also dedicate one section to those based upon heavily doped semiconductors. Finally, we discuss strategies involving site-selected growth for the rational fabrication of various types of hybrid nanostructures, with an emphasis on the experimental parameters that can be controlled to tailor the properties for a broad spectrum of applications.

## 2 Hot Electrons Involving Plasmonic Metals

Plasmon-induced hot electrons refer to the energetic electrons produced as a result of the optical excitation and then nonradiative decay of a plasmon. They are “hot” because they are not in thermal equilibrium with the crystal lattice of the host material.^19^ To put these energetic electrons to work for generating electrical energy or accelerating a chemical reaction, it is of critical importance to have a solid understanding of the fundamental mechanisms. To this end, theoretical calculations have offered an instrumental and insightful analysis and readers should refer to the review articles by Govorov^15^ and Atwater.^23^ Here we only provide a brief discussion on this topic in terms of general concepts and experimental variables, with a focus on plasmonic metals.

### 2.1 Generation of Hot Electrons

**Figure 2:**
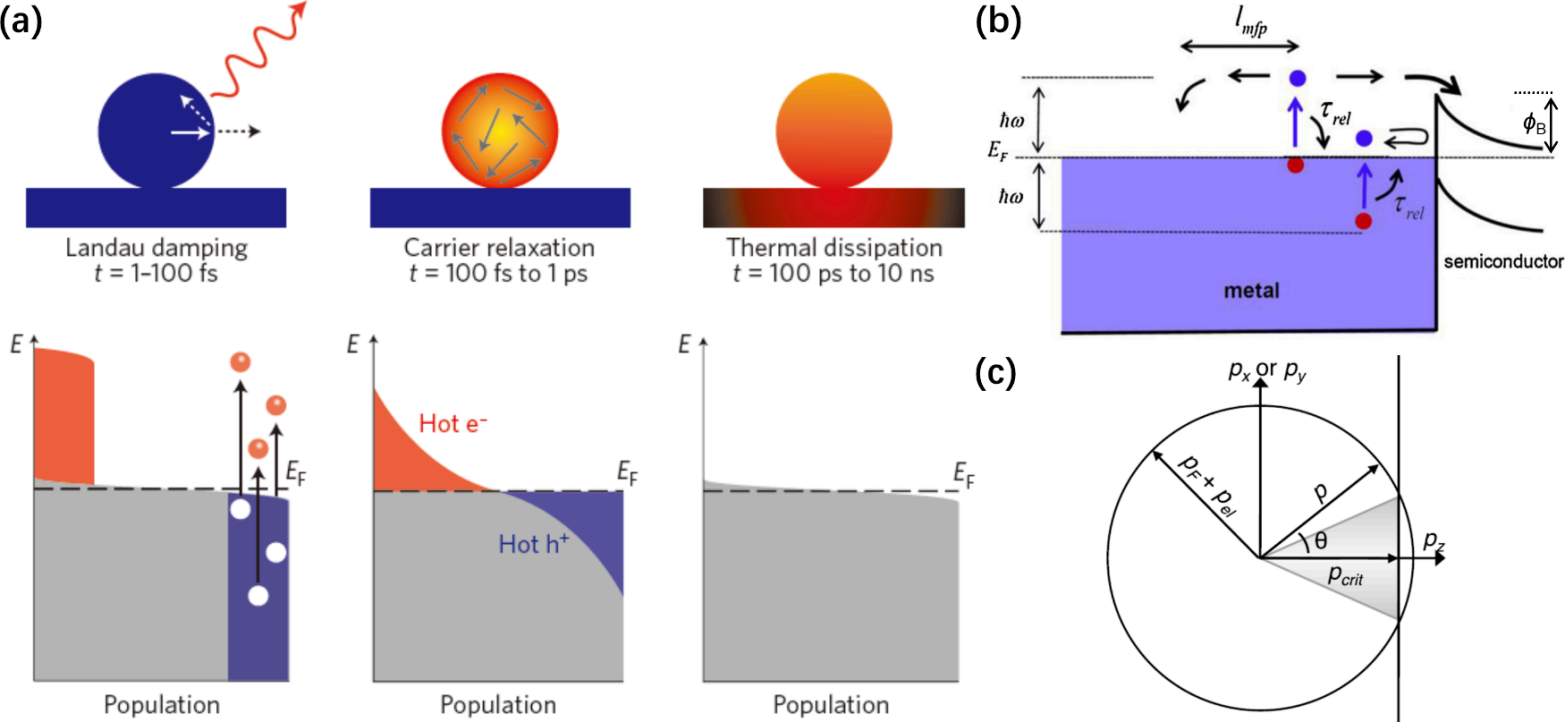
(a) Characteristic time scales and energy distributions involved in hot-electron generation. The Landau damping of LSPR and thus generation of hot electrons occurs on a time scale of 1–100 fs through electron–electron scattering. The relaxation of hot electrons through electron–phonon scattering is on a time scale of 100 fs to 1 ps, heating the lattice of the solid material. In comparison, thermal dissipation into the surrounding medium via phonon–phonon scattering takes place on a time scale of 100 ps to 10 ns. Reproduced with permission from ref (19). Copyright 2016 Springer Nature. (b) Hot-electron relaxation and injection into an adjacent semiconductor. Nonradiative decay of a plasmon with energy of ℏω produces hot electrons and holes through interband and intraband excitations. The hot electrons and holes would lose their energy within the relaxation time τ_rel_ or mean free path *l*_mfp_. A Schottky barrier of ϕ_B_ is generated at the interface between a metal and a semiconductor. The high-energy electrons have an opportunity to be injected into the conduction band of the semiconductor, whereas the low-energy electrons would be reflected back. Reproduced with permission from ref (15). Copyright 2014 Elsevier. (c) Momentum matching for hot-electron injection, in which the incident angle has to be within the “escape cone” (gray color) because the momentum normal to the interface must be greater than a critical value of *p*_crit_ = [2m*(*E*_F_ + ϕ_B_)]^1/2^. Reproduced with permission from ref (26). Copyright 2014 AIP Publishing.

The plasmon excited under light illumination can decay through both radiative and nonradiative channels. The radiative decay corresponds to elastic scattering and optical radiation on a time scale of ∼100 fs.^19^ In parallel, the nonradiative decay through Landau damping (i.e., electron–electron scattering) occurs on a time scale of 1–100 fs, as shown in Figure 2a, producing hot electrons with energies greater than that of the background thermal distribution. The energetic electrons are then converted to lattice heat through a slower process (100 fs to 1 ps) attributed to electron–phonon scattering. The subsequent heat dissipation into the environment via phonon–phonon scattering can last even longer than several hundred picoseconds. Before being converted to lattice heat, the hot electrons can also be collected and utilized by adding other components (e.g., molecular species and nanoparticles made of semiconductors or metals) to the surface of the plasmonic metal.^15,26^ The collection efficiency critically depends on the energy and momentum distributions of the hot electrons. Only those with proper distributions can be extracted (Figure 2, b and c).

The production rate of hot electrons, that is, the total number of hot electrons generated per unit of time and volume, is dominated by the localized field and absorption cross-section of LSPR excitation.^27^ In general, stronger LSPR leads to more hot electrons per unit time and volume. The energized electrons generated by plasmon excitation (ℏω) have energy distribution from the Fermi energy (*E*_F_) to the maximum value (*E*_F_ + ℏω). The energy distribution of hot electrons is also vital to their collection because those high in energy have a greater chance to overcome the interfacial barrier than the low-energy ones populated near the Fermi level.

#### 2.1.1 Dependence on the Geometric Parameters

**Figure 3:**
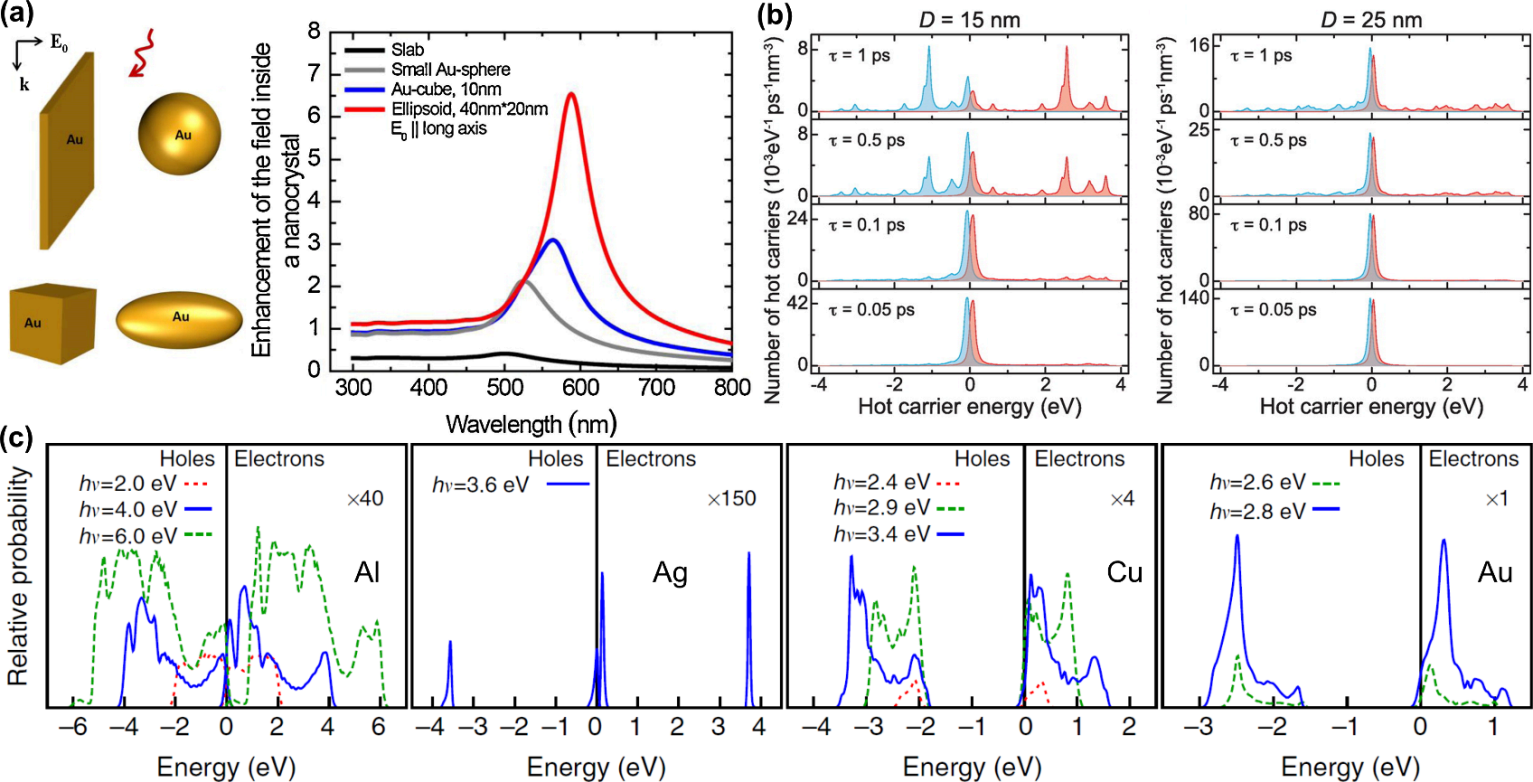
(a) Models of Au nanocrystals with different shapes and their calculated enhancement factors for the electric field inside the nanocrystals (suspended in water). Reproduced with permission from ref (15). Copyright 2014 Elsevier. (b) Energy distributions of hot electrons (red traces) and holes (blue traces) for two Ag nanospheres with diameters of 15 and 25 nm, respectively. Four different hot carrier lifetimes (τ) ranging from 0.05 to 1 ps are considered. The excitation photon energy is fixed at 3.65 eV, corresponding to the plasmon frequency. Zero energy refers to the Fermi level. Reproduced with permission from ref (27). Copyright 2014 American Chemical Society. (c) Energy distributions (relative to Fermi level at 0) of hot electrons (positive) and hot holes (negative) for various photon and plasmon energies, in Al, Ag, Cu and Au. (Note that the surface plasmon and the initial photon have the same energy, *hν*.) Reproduced with permission from ref (29). Copyright 2014 Springer Nature.

The geometric parameters of a plasmonic nanocrystal, including both shape and size, have major impacts on the energy and intensity of LSPR and thus the generation rate and energy distribution of hot electrons.^27−34^ As shown in Figure 3a for four types of Au nanocrystals, the calculated field enhancement has a strong dependence on the geometric shape. Since the generation rate of hot electrons is determined by the plasmon-enhanced electric field inside the nanocrystal, the ellipsoidal shape would be advantageous owing to the strong field enhancement associated with its longitudinal LSPR. For a specific shape, such as spherical, larger nanocrystals have stronger LSPR and thus generation of more hot electrons per unit time. However, the number of high-energy electrons will decrease as the particle size is increased due to the quantum confinement effect (Figure 3b).

#### 2.1.2 Dependence on the Material

In general, the energy distribution of hot electrons has a corresponding character related to the plasmonic metal. The plasmon decay through electron–electron scattering can excite the energetic electrons by intraband transitions (within the *sp* band) and interband transitions (from *d* band to *sp* band). The intraband transitions produce both energetic electrons and holes. The interband transitions produce mostly energetic holes and low-energy electrons near the Fermi level because the electrons are excited from the *d* band deep below the Fermi level (Figure 2b), which is related to the LSPR energy and interband energy determined by the electronic structure of the material (Table 1).^35−39^ As shown by the calculated energy distributions of hot electrons and holes in Figure 3c for a planar metal–dielectric interface,^29^ Al shows a continuous energy distribution ranging from zero to the plasmon energy for both hot electrons and holes; both high-energy electrons and high-energy holes are produced in Ag; and the predominant interband transitions in Au and Cu produce low-energy electrons and high-energy holes.

**Table 1:** Work Function, Interband Transition Energies, and Typical LSPR Peaks of Four Major Plasmonic Metals

Materials	Work function^35^ (eV)	Interband energy^36^ (eV)	Typical LSPR peak wavelength/peak energy (nm/eV)	Ref.
Au	5.1	1.8, 2.4	520/2.38 (7.2 nm in diameter, in water)^37^	(35−37)
Ag	4.26	3.8	393/3.16 (17.2 nm in diameter, in water)^37^	(35−37)
Cu	4.65	2.1	558/2.22 (10 nm in diameter, in water)^38^	(35, 36, 38)
Al	4.28	1.5	300/4.13 (70 nm in diameter, covered by an oxide shell, in air)^39^	(35, 36, 39)

### 2.2 Collection of Hot Electrons

The hot electrons can be extracted from plasmonic nanocrystals and utilized to drive photoelectric conversion processes or improve the energy conversion efficiency in an array of light-driven processes. However, the mismatch in time scale between the generation of hot carriers (fs to ns) and chemical reactions (ms to s) inevitably leads to low efficiency in utilizing plasmonic hot charge carriers. Additional dynamic processes are needed to extend the lifetimes of hot charge carriers and thus enable their effective participation in chemical reactions. To this end, a carefully designed hybrid nanostructure is necessary for achieving the efficient collection of hot electrons.

#### 2.2.1 Plasmonic and Catalytic Materials

By tailoring the size, shape, and morphology of plasmonic nanocrystals, it is feasible to obtain large plasmonic field enhancement and thereby generation of hot electrons at a reasonable rate and energy distribution as discussed above, in addition to spectral responses sought for catalytic applications. For the visible region, the commonly used plasmonic materials include Au, Ag, and Cu, whereas Al is only suitable for the ultraviolet (UV) region. Because of their susceptibility to sulfurization and oxidation, respectively, both Ag and Cu are not stable under ambient conditions. In comparison, Au exhibits the best stability and inertness in various environments, making it favorable for catalytic applications. Other nonmetallic materials such as heavily doped semiconductors also exhibit characteristic LSPR.

The plasmonic nanocrystals can be directly hybridized with molecules to collect the hot carriers and thereby initiate reduction and/or oxidation reactions. Alternatively, the plasmonic nanocrystals can be integrated with other functional materials, such as those featuring catalytic properties, to collect and utilize plasmon-induced hot electrons *via* interfacial energy and charge transfer. Notable examples include noble metals such as Pt, Pd, Ru, and Rh with superior catalytic activities toward an array of reactions. These metals are known for suffering from strong plasmon damping and thus lack of high-quality LSPR. When deposited on the surface of plasmonic nanocrystals, they can act as photocatalytic hot spots. Since the contact between plasmonic and catalytic metals is Ohmic in nature, which refers to a low resistance and low energy barrier at the interface, hot electron transfer is favorable. The other type of material is based on semiconductors. At the metal–semiconductor interface, a Schottky contact would be created along with an energy barrier. The Schottky contact requires that the energetic electrons have an adequate energy to overcome the barrier in order to be injected into the semiconductor (Figure 2b). In addition, the incident angle of the hot electrons should lie within the injection cone, otherwise, the electrons will be reflected back (Figure 2c).^26^ Taken together, only hot electrons with suitable energy and momentum distributions can be collected by an adjacent semiconductor. The Schottky barrier height is determined by the Fermi level of the metal and the band structure of the semiconductor. Meanwhile, the energy position of the semiconductor’s conduction band is pivotal to the catalytic reaction occurring on the surface of the semiconductor.

#### 2.2.2 Structural Features

During the transfer of hot electrons, they would be scattered by other electrons and/or the lattice, gradually losing their energy and the anisotropy of momentum distribution within a relaxation time of τ_rel_ or mean free path of *l*_mfp_. As a result, the temporal and spatial distributions of hot electrons also present a specific limitation on the collection efficiency. The scattering rate of hot electrons strongly depends on their energy, as well as their lifetimes ranging from 0.05–1 ps.^23,27^ These numbers suggest that the collection of hot electrons should be accomplished on an ultrafast time scale and over a very short distance. Taking Au as an example, when hot electrons of 1 eV are involved, it is possible to collect them within 100 nm and this distance will decrease to 10 nm for hot holes of 2 eV.^23^ The collection component should be deposited on the site coincided with the plasmonic hot spot featuring large local field enhancement. Therefore, the hot electrons can be transferred across the interface and injected into the collection component before losing their energy. The interface should be well organized with high quality, avoiding the scattering and trapping of hot electrons by defects. Additionally, the dimensions of the deposited catalytic component should be optimized, for example, to offer a large area of active surface and a short distance for the electrons to move from the interface to the outer surface.

#### 2.2.3 Coherent CID/PRET Processes and Other Mechanisms

Although hot electron transfer is the main focus of this article, other plasmon decay channels such as CID and PRET could directly pump the energized electrons to the excited states of the adjacent functional molecules or materials. These coherent processes also occur on an ultrafast time scale and need an extremely short distance between the two components. Meanwhile, both coherent CID and PRET can coexist with hot-electron transfer and contribute to the photochemical process. In general, hot-electron transfer can involve two mechanisms: indirect process *via* three-step electron transfer and direct process *via* one-step resonant excitation. These two mechanisms will be discussed later. Both the coherent CID/PRET processes and the direct transfer of hot electrons involve a strong coupling between the plasmon and the exciton in a molecule and semiconductor. The detailed physical mechanism for the hot electron collection *via* plasmon-exciton coupling is still under debate and calls for further studies.^40−47^

In the following sections, we highlight some exciting applications enabled by hot-electron injection, together with a brief discussion on the strategies and challenges for preparing the hybrid nanostructures.

## 3 Hot-Electron Transfer from a Plasmonic Metal to a Molecular Adsorbate

Hot electrons can be directly extracted from a plasmonic metal nanocrystal by molecular species adsorbed on its surface and further utilized to facilitate a chemical reaction. In a process referred to as plasmonic photocatalysis, the reaction is accelerated by bringing the reactant molecules into direct contact with plasmonic nanocrystals.^48,49^ In this case, the hot carriers can be directly used to reduce and/or oxidize the compounds involved. While the plasmon-mediated chemical reactions show many interesting features sought for light-harvesting applications, the mechanistic details for hot-electron-induced chemical reactions are still under debate.^48−53^

Typically, hot-electron transfer between a plasmonic metal and a molecular adsorbate involves three major steps: plasmon decay, hot-electron generation *via* electron–electron scattering, and hot-electron injection. The energized electrons injected into the molecule could activate the rate-limiting step at a lower temperature compared to the conventional catalyst. This three-step process is referred to as an indirect process, and its efficiency is often low because there are too many steps involved and the decay of hot electrons occurs on a very fast (ps) time scale. Alternatively, recent studies proposed a direct mechanism for hot-electron transfer, where the decay of a plasmon excites a hot electron in the molecule strongly coupled to the plasmonic nanocrystal. This direct transfer process is ultrafast (tens of fs, on par with the time scale of plasmon damping) and considered to be highly efficient. In addition, the plasmonic photothermal effect (another process arising from hot-electron generation) can also contribute to the acceleration of a catalytic reaction. How to separate the thermal effect from hot-electron-induced reduction in activation barrier plays an important role in advancing our understanding of plasmonic photocatalysis.

### 3.1 Indirect Transfer of Hot Electrons

**Figure 4:**
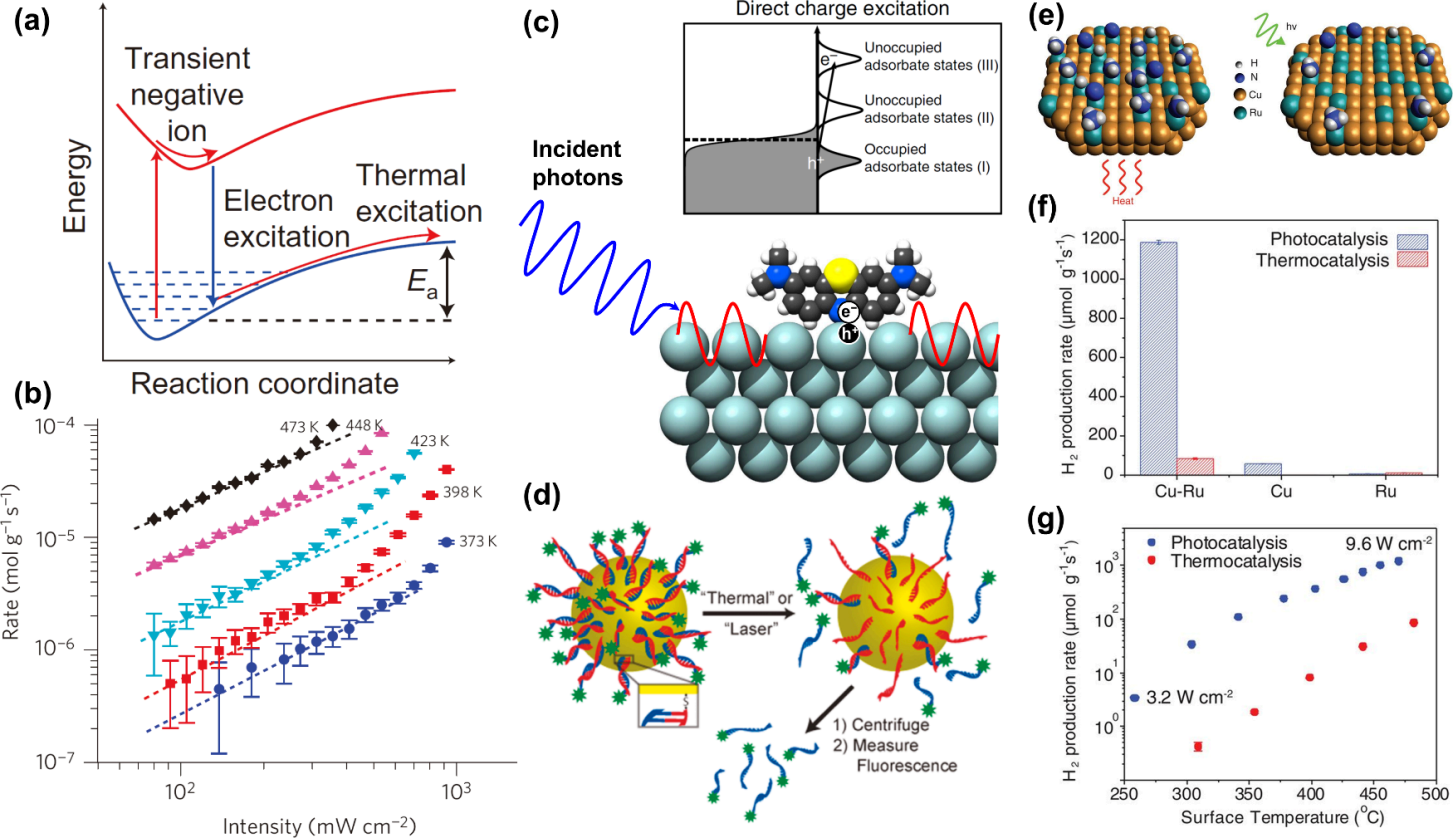
(a) Proposed mechanism of O_2_ dissociation assisted by hot-electron transfer. Inelastic scattering of hot electrons helps O_2_ molecules overcome the dissociation energy barrier *E*_a_. For comparison, the thermal activation pathway is also shown. Reproduced with permission from ref (54). Copyright 2011 Springer Nature. (b) A superlinear dependence of the photocatalytic rate of ethylene epoxidation (limited by the dissociation of O_2_) as a function of light intensity at various temperatures. Reproduced with permission from ref (58). Copyright 2012 Springer Nature. (c) Direct hot-electron transfer for selective chemical reaction. The plasmon decay directly results in electron excitation into a high-energy, unoccupied adsorbate orbital (III) that matches the plasmon energy, opening the possibility for selective reaction path that is impossible through the indirect mechanism. Reproduced with permission from ref (59). Copyright 2016 Springer Nature. (d) Thermal and light-triggered release of DNA molecules from Au nanoshells. Upon heating or laser illumination, the cargo with a complementary sequence (blue) and tagged with fluorescein molecules (green) was released from the thiolated host sequence (red) attached to the Au surface. Reproduced with permission from ref (61). Copyright 2011 American Chemical Society. (e) Catalytic ammonia decomposition due to photocatalysis (right) enabled by hot-electron transfer, which promotes desorption of adsorbed intermediates relative to thermocatalysis (left) by a Cu–Ru alloy nanocrystal consisting of light-harvesting antenna in the form of Cu nanoparticle and Ru reactor sites on the surface. (f) H_2_ formation rate of photocatalysis (9.6 W·cm^–2^) and thermocatalysis (482 °C) on Cu–Ru, Cu, and Ru nanoparticles. (g) Comparison of photocatalytic and thermocatalytic rates on Cu–Ru nanoparticles. The horizontal axis corresponds to the surface temperature caused by photothermal heating (photocatalysis) or external heating (thermocatalysis). Reproduced with permission from ref (62). Copyright 2018 AAAS.

When the energy barrier of the rate-limiting step is relatively high, the chemical reaction is usually conducted at an elevated temperature in order to achieve a reasonable reaction rate through thermal activation. However, the elevation of operating temperature may bring in many adverse impacts, in addition to the demand on energy. The introduction of a proper catalytic material can reduce the energy barrier, increasing the reaction rate without involving elevation in temperature. In the case of plasmonic photocatalysis, particularly, the plasmon-induced hot electrons can be leveraged to drive catalytic reactions at temperatures significantly lower than those typical of the conventional processes. To this end, Linic and co-workers demonstrated a plasmonic photocatalyst based on 60 nm Ag nanocubes to accelerate the epoxidation of ethylene under the irradiation of visible light.^54^ Their results implied that the participation of hot electrons increased the overall reaction rate through an electron-assisted O_2_ dissociation mechanism. It was proposed that an energetic hot electron was transferred from the Ag nanocube to the antibonding O–O 2π* state of an adsorbed O_2_, resulting in the formation of a transient O_2_^–^ anion (Figure 4a). The lifetime of the excited molecules on metal surface was 5–10 fs, which was not long enough to dissociate O_2_. When the electron was transferred back to Ag, energy was deposited into the O–O vibrational mode through inelastic scattering of hot electron, facilitating the dissociation of O_2_. The average time for the dissipation of vibrational energy is typically on the order of picoseconds, exceeding the oscillation time for vibration of O_2_, and the molecule has enough time to react.

Compared to the thermal activation process, plasmon-induced hot-electron transfer can activate a chemical bond at a significantly lower temperature and under a low flux of photons (on the order of solar intensity).^55−57^ Furthermore, a transition from the linear to superlinear power law dependence was observed for the photocatalytic reaction rate and light intensity when irradiated by light with an intensity ∼10^9^ times lower than what is required for the observation of superlinear behavior on a bulk metal surface (Figure 4b).^58^ This unique characteristic can be attributed to the inelastic scattering of hot electrons for bond activation, accompanied by plasmonic local field enhancement and plasmon-induced elastic photon scattering with improved light absorption length.

### 3.2 Direct Transfer of Hot Electrons

The conventional hot-electron collection process involves three steps and such an indirect process often has a low efficiency because hot-electron transfer requires interfacial charge separation on an ultrafast time scale.^19^ When studying the plasmon-enhanced photocatalysis of methylene blue (MB) on Ag nanocubes, Linic and co-workers also proposed a direct hot-electron transfer process.^59^ They investigated this process by integrating wavelength-dependent surface-enhanced Raman scattering (SERS) characterization with kinetic analysis of the photocatalytic reaction rate. It was observed that the ratio of anti-Stokes to Stokes signals for the Ag-MB complexes at 785 nm excitation was much higher than that at 532 nm, greatly exceeding the values derived using Boltzmann distribution. The change in MB signal (due to desorption and/or decomposition) under exposure to the 785 nm laser was significantly larger (4.8 times at *t* ≈ 0) than that at 532 nm excitation under the same light intensity. It was suggested that the plasmon decay induced the direct and resonant excitation of hot electrons to the specific hybridized states of the molecules adsorbed on the Ag nanocrystals,^60^ activating the photochemical transformation (desorption and/or decomposition). The one-step, direct charge transfer was more efficient than the conventional three-step process. Moreover, this resonant process can potentially pump the energetic electrons to the high-energy molecular states matching the resonant energy condition (Figure 4c). In principle, the direct hot-electron transfer can be used to drive a selective chemical pathway that is ineffective or impractical for the indirect mechanism. In contrast, the three-step mechanism preferentially proceeds through an activation pathway by means of lower-energy molecular states because a large proportion of the hot electrons are distributed near the Fermi level.

### 3.3 Hot-Electron Transfer versus Photothermal Heating

As two competing channels with different time scales in a plasmon decay process (Figure 2a), hot-electron transfer and photothermal heating can both contribute to the acceleration of a chemical reaction. It is well-known that heating increases the kinetic energy of molecular species and thus their reactivity. However, how to separate the impacts from photothermal heating and hot electron transfer on a chemical reaction has been a challenge because these two effects typically occur simultaneously. Furthermore, while an elevated surface temperature can increase electron transfer kinetics, there is still no direct evidence to support the argument that photothermal heating would influence hot electron transfer. In one study, Halas and co-workers demonstrated that coupling hot-electron transfer with photothermal heating can induce the release of DNA from Au nanostructures such as nanoshells or nanorods. Through the dehybridization of a double-stranded DNA, single-stranded DNA tagged with a fluorescein “cargo” could be released from the complementary thiolated, single-stranded DNA “host”, which remained strongly bound to the Au surface (Figure 4d).^61^ Their results indicated that approximately 20% of the DNA released from the nanoshell-DNA sample was caused by the hot-electron transfer occurring below the DNA melting temperature, while the photothermal effect was responsible for all the DNA released from the nanorod-DNA sample *via* DNA melting at a high temperature. In literature, although experiments were typically performed under temperature control to exclude the photothermal effect from hot-electron transfer, there is always controversy because there exists a difference between the equilibrium temperature in solution and the nonequilibrium, local temperature on the surface of a plasmonic nanocrystal. In this regard, Nordlander, Halas, and co-workers reported a method to quantify the contributions from either hot-electron transfer or photothermal heating in a plasmonic photocatalysis involving the decomposition of ammonia into N_2_ and H_2_ (Figure 4e).^62^ The plasmonic antenna-reactor photocatalyst was a Cu–Ru alloy surface consisting of a Cu nanoparticle antenna and Ru reactor sites (the concept of antenna-reactor will be discussed in Section 5.2). The hot-electron transfer modifies the reaction kinetics by reducing the activation barrier to the rate determining step corresponding to N_2_ desorption. The photocatalytic reaction rate on the Cu–Ru alloy surface was about 20 and 177 times of those on the pure Cu and Ru nanoparticles, respectively (Figure 4f). The surface temperature of the photocatalyst was measured using a thermal imaging camera to account for the photothermal effect. Their analysis suggested that the thermocatalytic rate corresponding to the production of H_2_ by photothermal heating was 1–2 orders of magnitude lower than the observed photocatalytic rate derived from hot-electron transfer (Figure 4g).^62^ In another study, Huang and co-workers demonstrated that photothermal heating made the most important contribution to light-driven catalytic hydrogenation. By controlling the shell thickness of Au@Pd core–shell nanorods at the atomic level, the authors demonstrated that the supply of hot electrons to Pd surface affected Pd–H dissociation adversely and thereby reduced hydrogenation efficiency, while photothermal heating contributed positively to hydrogenation reactions.^63^

## 4 Hot-Electron Transfer from a Plasmonic Metal to a Semiconductor

For photocatalysis relying on the direct interactions between plasmonic nanocrystals and reactant molecules, a major obstacle is that the surface of a plasmonic metal (e.g., Au, Ag, Cu, or Al) is often inactive toward the reaction of interest. Semiconductors have excellent optical and electronic properties, as well as remarkable chemical and catalytic activities. However, the light absorption capability of a semiconductor is often limited by its bandgap, with TiO_2_, for instance, being only sensitive to UV light. Compared with those made of plasmonic metals, semiconductor nanocrystals have relatively small absorption/scattering cross sections (Figure 1a). As such, integrating metals with semiconductors offers a viable system based on metal–semiconductor heteronanostructures for the development of light-driven devices.

The concentration of electrons in an n-type semiconductor can be increased by injecting the hot electrons from a plasmonic metal. The contact between a metal and a semiconductor usually generates a Schottky barrier, which affects the injection efficiency of the hot electrons. The barrier height is dependent on the Fermi level of the metal and the electronic band structure of the semiconductor. The energy of hot electrons is determined by the shape, size, and composition of the plasmonic nanostructure, which is typically located in the range of 0–4 eV for Au, Ag and Cu (Figure 3c). The energized hot electron can overcome the Schottky barrier if the semiconductor is selected with an appropriate band structure. Meanwhile, the density of states in the conduction band determines the electron trapping ability, and the position of the conduction band is crucial for the photochemical reaction occurring on the surface of the semiconductor. The semiconductor component should be placed in the plasmonic hot spot for the effective injection of hot electrons. Therefore, design and fabrication of the metal–semiconductor hybrid structure is a key step toward the realization of light-driven devices and related applications. The integration of plasmonic metals with elemental semiconductors is often achieved through a top-down approach by leveraging the mature fabrication technology developed for silicon and germanium. In this case, the size, shape, and arrangement of the hybrid system can be precisely controlled. For the oxide and chalcogenide semiconductors, a bottom-up approach based on colloidal synthesis has been developed in recent years. In this section, we discuss how to extract and utilize hot electrons *via* the metal–semiconductor interface in the context of elemental semiconductors, oxides, chalcogenides, and 2D materials, respectively.

### 4.1 Elemental Semiconductors

**Figure 5:**
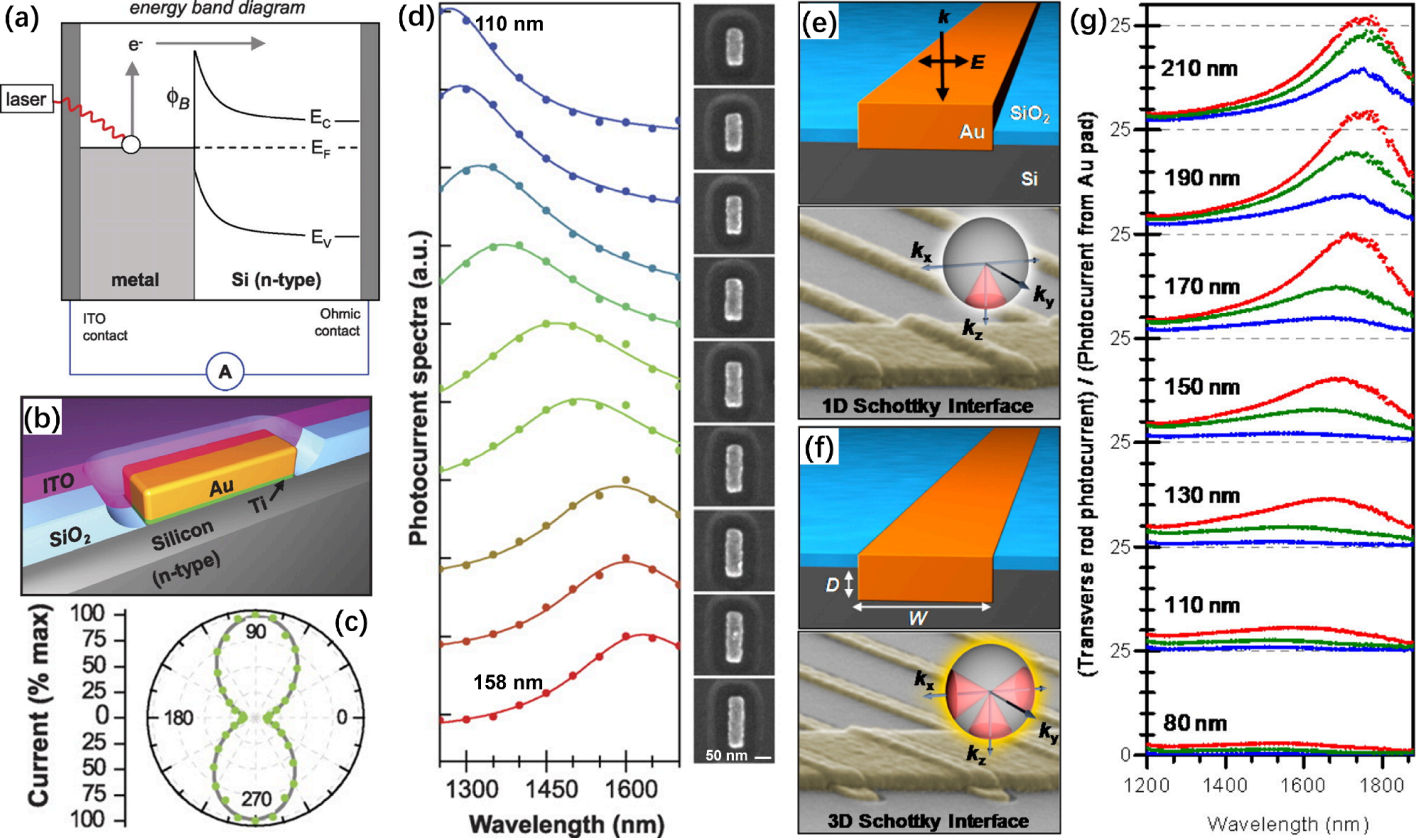
(a) Band diagram of nanoantenna-semiconductor photodetection with a Schottky barrier (ϕ_B_). (b) Representation of a single rectangular Au nanorod antenna on an n-type Si substrate. (c) Polarization dependence of photocurrent with an angular dependence of cos^2^θ. (d) Experimental photocurrent spectra for Au antennas with nine different lengths from 110 to 158 nm (from top to bottom). All the antennas were 50 nm wide and 30 nm thick. Reproduced with permission from ref (3). Copyright 2011 AAAS. (e) A planar device that only supports electron transport through the bottom interface. (f) A fully embedded device that supports electron transport through all three Schottky interfaces. Electrons can only emit across the Schottky junction when their *k*-vector lies inside the emission cone and their energy exceeds the Schottky barrier. (g) Photocurrent spectra measured for the devices with width varying from 80–210 nm, while being embedded 5 (blue), 15 (green), and 25 nm (red) in the Si substrate. Reproduced with permission from ref (69). Copyright 2013 American Chemical Society.

A top-down approach based on semiconductor device fabrication has been successfully used to integrate plasmonic metals with elemental semiconductors (e.g., silicon), and it typically involves multiple steps of lithographic and chemical etching processes. In one example, Au nanostructures with high stability have been combined with Si for the fabrication of photodetectors.^64−68^ The contact between Au and n-type Si creates a Schottky barrier and the plasmon-induced hot electrons can be collected and detected by a Si-based electric circuit. The generation of photocurrent is no longer limited by the bandgap of the semiconductor because the metallic nanostructure has size- and shape-dependent optical responses, together with a large absorption cross-section. Halas and co-workers reported a photodetection device based on an active optical antenna in the form of an array of rectangular Au nanorods on n-type Si (Figure 5, a and b).^3^ Since the Au nanorods support size-dependent longitudinal and transverse LSPRs, this photodetection device displayed resonant and polarized detection characteristics, with highly tunable spectral responsivity dependent on the geometric parameters of the Au nanorods (Figure 5, c and d). To meet the momentum requirement for hot-electron injection, they also demonstrated a 3D configuration for the Schottky interface by embedding Au nanorods in Si substrates to increase the hot-electron injection efficiency. They observed a 25-fold increase in efficiency than the counterpart device based on a planar Schottky contact (Figure 5, e-g).^69^

### 4.2 Oxide Semiconductors

As the most frequently used oxide semiconductor, TiO_2_ has a wide bandgap (*E*_g_ = 3.3 eV), excellent chemical stability, high activity, and superb electron-accepting capability because of the high density of states in its conduction band.^70,71^ A range of applications have been demonstrated with TiO_2_ nanocrystals, including photocatalytic water splitting and dye-sensitized solar cells.^72−74^ The plasmon-induced transfer of hot electrons provides a new avenue for the augmentation of these applications.^48−50^ Beyond TiO_2_, other oxide semiconductors, such as ZnO,^75−77^ CeO_2_,^78,79^ WO_3_,^80,81^ and Cu_2_O,^82^ have all been investigated to produce plasmon-induced hot electrons for photovoltaic and photocatalytic applications. In this section, we focus on the utilization of hot electrons in terms of plasmonic photosensitization, plasmonic artificial photosynthesis, and interfacial engineering.

#### 4.2.1 Plasmonic Absorber and Photosensitizer

The utilization of carriers photoexcited in TiO_2_ was initially demonstrated for photoelectrochemical water splitting under UV radiation by Fujishima and Honda.^72^ Due to its wide bandgap, however, such a device cannot be operated under visible light. A significant breakthrough was made by Grätzel and co-workers in the development of dye-sensitized solar cells,^73,74^ in which dye molecules with intense and flexible light-harvesting capability were incorporated to absorb light and collect energy through electron transfer from the dye molecules to TiO_2_. Similarly, plasmon-sensitized solar cells and plasmonic photovoltaics have been fabricated by employing plasmonic nanocrystals as light-harvesting materials to replace the conventional light absorbers based on dye molecules and semiconductors.^83,84^ Built upon the Au/TiO_2_ heterojunction, many studies have demonstrated the light-harvesting capability of Au nanocrystals and the effect of hot-electron injection from plasmonic nanocrystals to the conduction band of TiO_2_ in photocatalytic reactors, solar cells, and photodetective devices, with greatly improved energy conversion efficiency.^85,86^

#### 4.2.2 Artificial Plasmonic Photosynthesis

**Figure 6:**
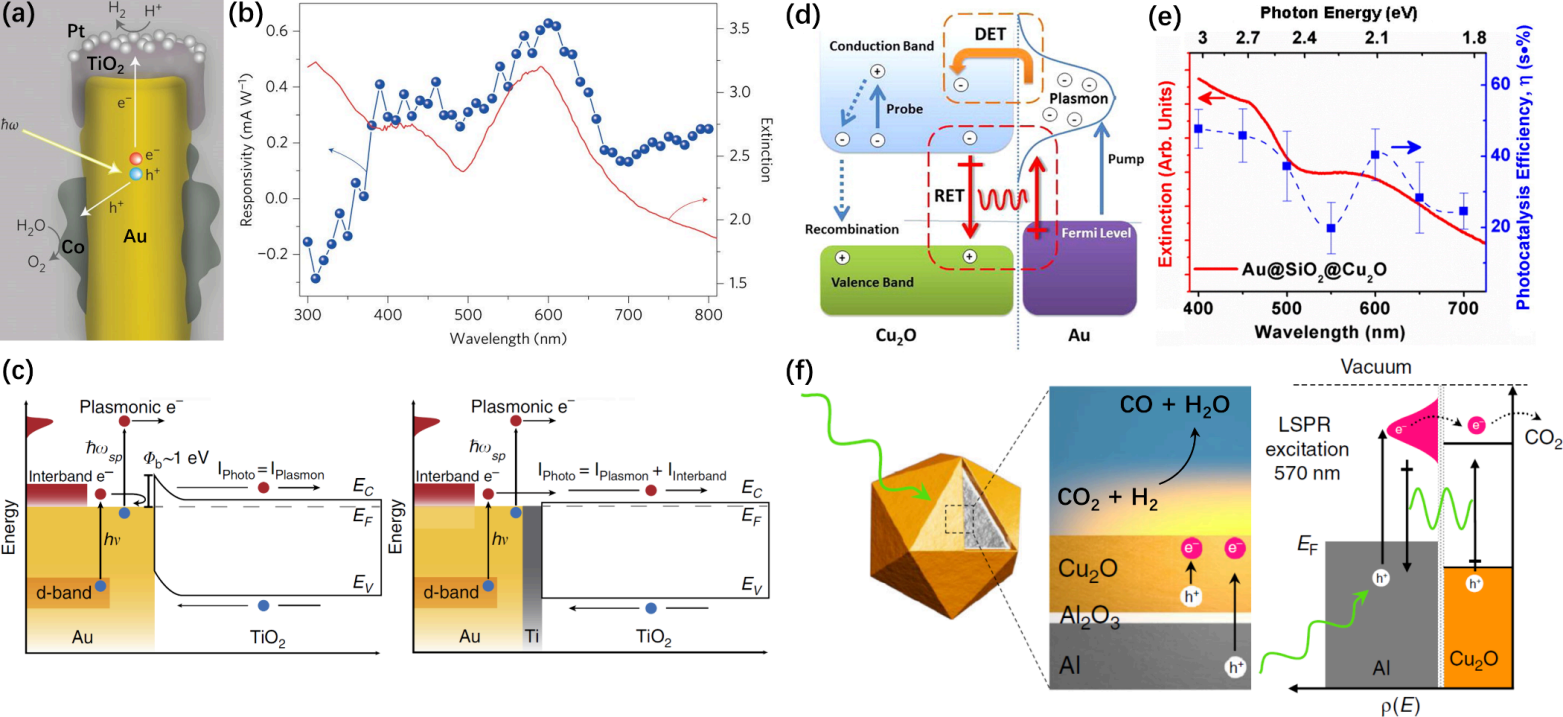
(a) Schematic of an individual photosynthetic unit showing the Au nanorod, the TiO_2_ cap decorated with Pt nanoparticles as a hydrogen evolution catalyst, and the Co nanoparticles as an oxygen evolution catalyst. (b) The measured photocurrent spectrum of the Au/TiO_2_/Pt plasmonic photocathode (blue symbols) matches the extinction spectrum of the device (red line). Reproduced with permission from ref (87). Copyright 2013 Springer Nature. (c) Band diagrams showing a Schottky device made of Au/TiO_2_ and an Ohmic device made of Au/Ti/TiO_2_. Reproduced with permission from ref (90). Copyright 2015 Springer Nature. (d) Schematic representation of direct electron transfer (DET) and plasmon-induced resonant energy transfer (PRET) mechanisms that can occur in the Au@Cu_2_O structure. (e) The photocatalytic action spectrum generally follows the extinction spectrum of the Au@SiO_2_@Cu_2_O photocatalyst. The DET is suppressed by the SiO_2_ insulator. Reproduced with permission from ref (40). Copyright 2012 American Chemical Society. (f) Structure and mechanism of plasmon-assisted reverse water–gas shift (rWGS) reaction by the Al@Al_2_O_3_@Cu_2_O antenna–reactor with PRET and tunneling of hot electrons. Reproduced with permission from ref (91). Copyright 2017 Springer Nature.

Usually, it is difficult to promote reduction and oxidation reactions at the same time in a plasmon-mediated photocatalysis. Sacrificial reagents providing either electrons or holes can be introduced to facilitate the overall reaction. Moskovits and co-workers designed and tested a photosynthetic device to realize both oxygen evolution and hydrogen evolution reactions for water splitting.^87^ In this device, both hot electrons and hot holes were utilized. The device was constructed from an array of Au nanorods by coating one of their ends with a TiO_2_ layer, followed by the deposition of Pt nanoparticles on the TiO_2_ layer and Co nanoparticles on the exposed Au surface (Figure 6a). The Au nanorods acted as the light-harvesting component. After the light-excited plasmons had decayed into hot electrons, the energetic electrons capable of overcoming the Schottky barrier were injected into TiO_2_. The electrons were finally transferred to the reduction catalyst made of Pt nanoparticles, participating in the hydrogen evolution reaction on the Pt surface. Spectral response measurements clearly demonstrated that LSPR played a key role in light harvesting because TiO_2_ is an optical material with UV sensitivity only (Figure 6b). On the other hand, the Co nanoparticles served as a catalyst toward the oxygen evolution reaction and they could collect hot holes to accelerate the reaction. To drive the overall redox reaction effectively, it is imperative to employ a rationally design structure that optimizes the interaction between the catalyst and the reactants.^88,89^ Interestingly, on the surface of Ag nanoparticles, accompanying the hot-electron-mediated reduction reaction, the oxidation reaction can also be achieved through a photoinduced dissociation of silver halides.^52^ This process serves to balance the presence of hot holes and thereby promotes the overall redox reaction.

#### 4.2.3 Interfacial Engineering

The existence of Schottky barrier implies that only those hot electrons with kinetic energies higher than the barrier can be injected into the semiconductor. As such, the low-energy hot electrons do not contribute to the energy conversion efficiency. Halas and co-workers found that the introduction of a 2 nm Ti layer was able to create an Ohmic contact between Au nanowires and a TiO_2_ substrate (Figure 6c).^90^ The absence of a barrier in the Ohmic contact allowed the low-energy electrons, especially the photoexcited electrons induced by the interband transition from the *d*-band deep below the Fermi level, to be injected into the semiconductor, contributing to the energy conversion efficiency.

The hot-electron transfer will be blocked if there is an insulating layer between the metal and the semiconductor. This situation is unavoidable in some cases due to the formation of native oxides, for example, the ultrathin aluminum oxide layer on the surface of Al nanocrystals. In addressing this issue, other mechanisms, such as PRET^40−46^ and electron tunneling effect,^91−96^ have been utilized to pump energetic electrons into semiconductors. Wu and co-workers presented a PRET process to transport the energy stored in the plasmons of a metal core to the semiconductor shell in the Au@SiO_2_@Cu_2_O double-shelled nanoparticles.^40,41^ The SiO_2_ interlayer was added to exclude the direct electron transfer between Au and Cu_2_O (Figure 6d). The photocatalytic action spectrum for the photodegradation of methyl orange manifested the contribution of plasmon absorption around 600 nm and the energy transfer *via* a PRET process (Figure 6e). Halas and co-workers demonstrated plasmon-induced selective conversion of CO_2_ to CO using the earth-abundant Al@Cu_2_O core@shell nanoparticles. In this case, Al nanocrystals were wrapped with a 2–4 nm shell of amorphous Al_2_O_3_, preventing the direct transfer of electrons. In addition to the plasmonic local field enhancement and PRET effect, tunneling of hot electrons from the Al plasmonic antenna to the Cu_2_O catalytic reactor might also contribute to the photocatalytic activity (Figure 6f).^91^

### 4.3 Chalcogenide Semiconductors

Chalcogenide (sulfide, selenide, and telluride) nanocrystals, such as CdS, ZnS, CdSe, and CdTe quantum dots, are excellent light absorption and emission nanomaterials with tunable bandgap energies from visible to NIR region.^97^ Their bandgap energy could be easily tuned to resonance with LSPRs, leading to strong coupling with unique optical responses. In this section, we discuss electron transfer with tunable direction, in addition to the direct transfer mechanism for hot electrons. The 2D dichalcogenides are presented in the next section.

#### 4.3.1 Electron Transfer with Tunable Direction

**Figure 7:**
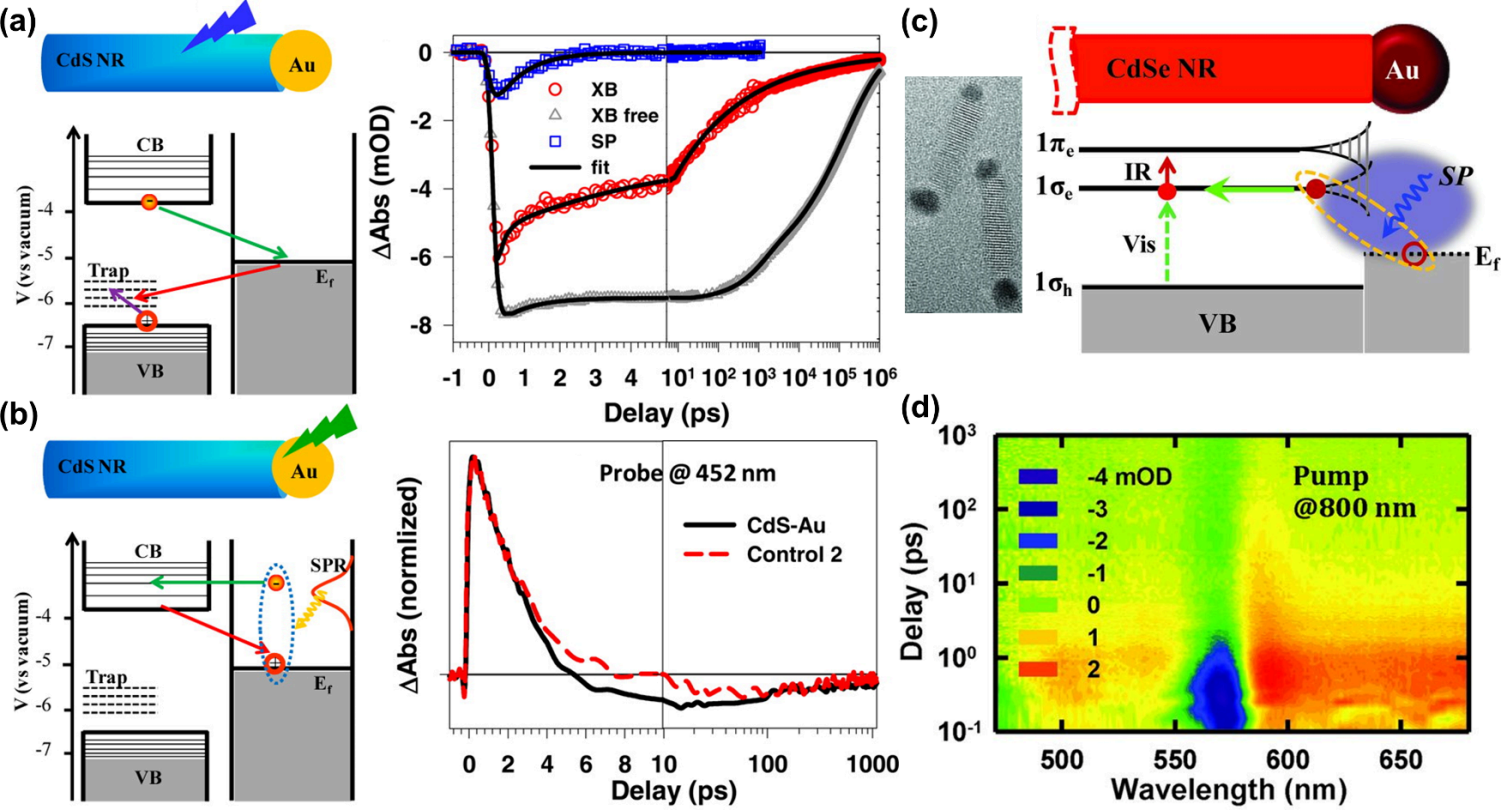
(a) Charge separation in a CdS–Au nanorod by exciton excitation in CdS pumped at 400 nm (green arrow: charge separation, red arrow: recombination, purple arrow: hole trapping). The transient absorption kinetics indicates that the 1Σ exciton bleach (XB) recovery probed at ∼450 nm in CdS–Au nanorods (red circles) is much faster than that (XB free) in free CdS nanorods (gray triangles) due to electron transfer from CdS to Au. The blue squares (SP) correspond to the kinetics of plasmon bleach of Au tip. (b) Charge separation in a CdS–Au nanorod by plasmon excitation in Au pumped at 590 nm (yellow arrow: nonradiative plasmon decay). The transient absorption kinetics probed at 452 nm for CdS–Au nanorods (black solid line) shows a 1Σ exciton bleach (ΔAbs <0) and noticeable difference from the control sample (a mixture of CdS nanorods and Au nanoparticles), attributed to hot-electron transfer from Au tip to CdS. Reproduced with permission from ref (98). Copyright 2013 American Chemical Society. (c) Schematic of the electronic structure and HRETM image of the CdSe–Au nanorod. The plasmon decay directly creates an electron in the conduction band of CdSe and a hole in Au. (d) 2D pseudocolor plot of transient absorption spectra of the CdSe–Au nanorods at 800 nm excitation show a pronounced 1Σ-exciton-bleach feature at ∼575 nm (ΔAbs with negative mOD) with a formation time of 20 ± 10 fs, corresponding to hot-electron transfer. Reproduced with permission from ref (99). Copyright 2015 AAAS.

The interaction between a plasmonic nanocrystal and a chalcogenide semiconductor can be manipulated by controlling the energy of the incident light. Lian and co-workers carefully investigated a tunable electron transfer process in a heteronanostructure consisting of a Au nanoparticle attached to one end of a CdS nanorod (Figure 7, a and b).^98^ The CdS nanorods show typical excitonic transitions at 452 nm (1Σ exciton) and 401 nm (1Π exciton) that are well-separated from the plasmon band of Au at 533 nm, allowing for selective excitation of the metal or semiconductor domains. Tuning the direction of electron transfer (from Au to CdS or *vice versa*) across the Au/CdS interface can be achieved by adjusting the excitation wavelength (e.g., 590 or 400 nm) and validated using transient absorption spectroscopy. Under excitation at 590 nm, excited plasmons in the Au tip induced hot-electron transfer from Au to CdS, resulting in a bleach of 1Σ band in CdS (probed at 452 nm, Figure 7b). The ultrafast hot-electron injection into CdS showed a quantum yield of ∼2.75% and a short-lived charge-separated state (with electrons in CdS and holes in Au) of 1.8 ps. On the contrary, under the excitation of 400 nm, excitons generated in the CdS nanorod were manifested as a bleach in the transient absorption spectroscopy (Figure 7a). The 1Σ exciton bleach recovery was accelerated by the electron or energy transfer from CdS to Au with a half-life of 4.8 ps, which led to a long-lived charge-separated intermediate with a half-life of 1.5 μs.

#### 4.3.2 Direct Transfer of Hot Electrons

The direct transfer mechanism discussed in Section 3.2 has also been applied to the metal–semiconductor interface. Lian and co-workers proposed a new mechanism for the interfacial generation of hot electrons, called plasmon-induced interfacial charge-transfer transition (PICTT), in a strongly coupled Au–CdSe hybrid with a Au tip on one end of the CdSe nanorod (Figure 7c).^99^ In the PICTT pathway, the nonradiative plasmon decay directly excites an electron in CdSe and a hole in Au, which are confined to the interfacial region. The transient absorption spectra of CdSe–Au nanorods under 800 nm excitation (Figure 7d) showed a pronounced bleach feature of 1Σ exciton at ∼575 nm, indicating the formation of CdSe conduction-band electrons through the PICTT process, which involves the direct excitation of a hot electron in CdSe by plasmon decay, accompanied with the creation of a hole in Au. Compared to the conventional hot-electron process, which involves three steps,^100^ the quantum efficiency of the one-step PICTT process is much higher (>24%). The direct transfer of hot electrons requires a strong coupling between the plasmonic material and the semiconductor. The strong plasmon–exciton coupling would produce many interesting phenomena, such as Fano interference and Rabi splitting,^47^ as well as the PRET effect discussed in Section 4.2.3. This strongly coupled plasmon–exciton hybrid is an attractive system with great potential for solar energy conversion.^101^

### 4.4 2D Semiconductors

2D semiconductors such as graphene and transition metal dichalcogenides have intriguing properties due to their single-atom thicknesses. The LSPRs exhibit a feature of evanescent fields on the nanoscale, together with large local field confinement and enhancement. These characteristics augment energy transfer and increase the rate of hot electron generation, thereby enhancing the efficiency of hot electron injection. When 2D materials are coupled to plasmonic nanocrystals, a large coupling strength is expected. This is because the intrinsic features of 2D morphology and single-atom thickness easily generate large interfacial areas and effective interaction volumes.^102−108^

#### 4.4.1 Plasmon-Induced Doping

**Figure 8:**
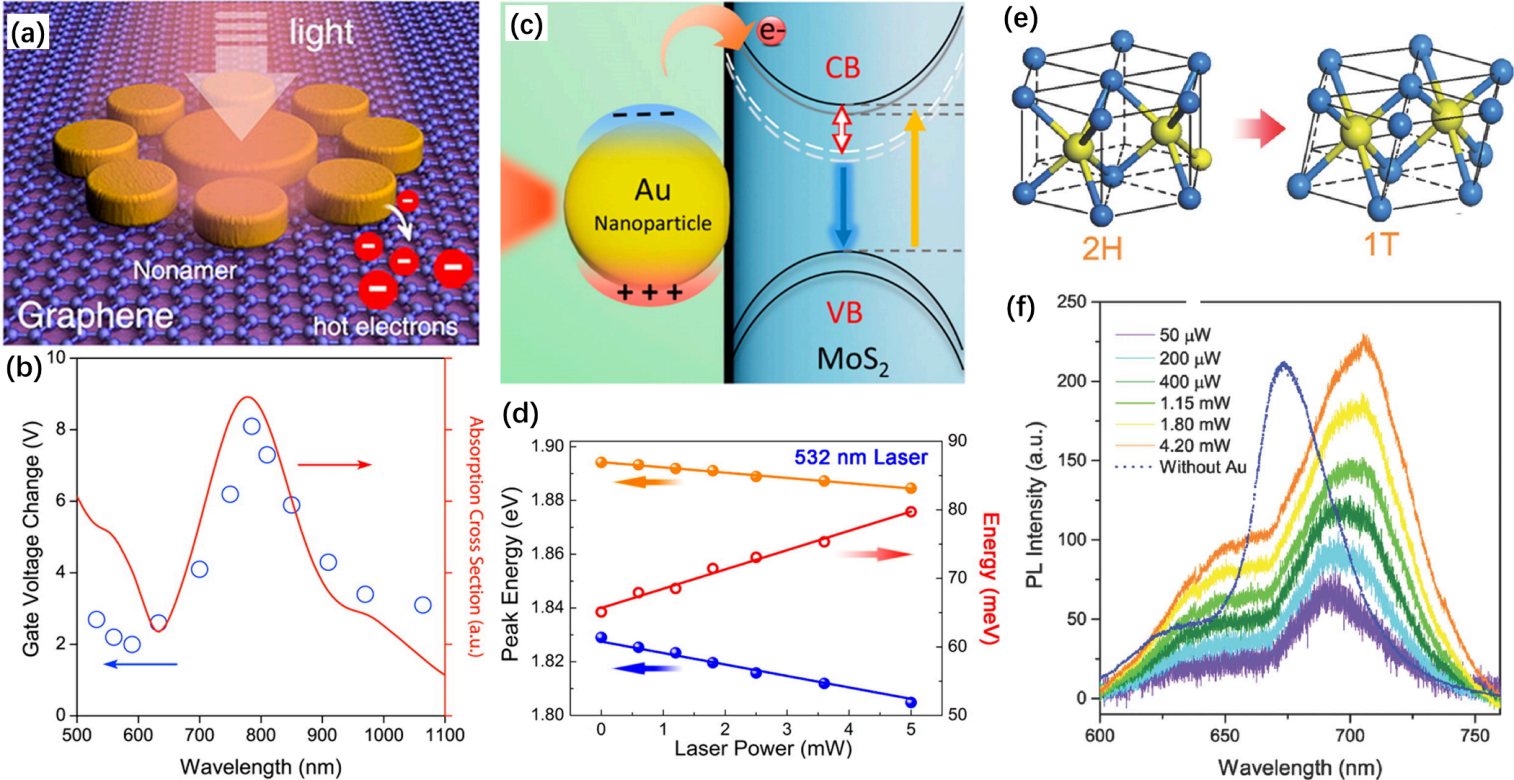
(a) Illustration of hot-electron injection from optically excited Au nonamer, causing n-doping to the underlying graphene sheet. (b) Plot showing Dirac voltage shift (blue circles) under different excitation lasers (extracted by comparing the Dirac voltage in the case without laser excitation) matches the simulated absorption cross-section of Au nonamer (red curve). Reproduced with permission from ref (109). Copyright 2012 American Chemical Society. (c) Schematic showing the interaction between a Au nanoparticle and a MoS_2_ monolayer. The white dashed line represents excitonic energy level. The yellow and blue solid arrows correspond to absorption and luminescence, respectively. The red hollow arrow represents the tuning of exciton binding energy due to n-doping *via* hot-electron transfer. (d) Absorption peak (yellow) and luminescence peak (blue) as a function of laser power. The red line is the energy difference between absorption and luminescence peaks, corresponding to the exciton binding energy. Reproduced with permission from ref (110). Copyright 2015 American Chemical Society. (e) Phase transition between the 2H and 1T lattice structures of MoS_2_. The yellow and cyan spheres represent Mo and S atoms, respectively. (f) Photoluminescence shift of MoS_2_ after Au deposition with incident powers ranging from 50 μW to 4.2 mW. The spectral shift is caused by the narrowing band gap associated with the 2H-to-1T transition of MoS_2_ monolayer. Reproduced with permission from ref (111). Copyright 2014 Wiley-VCH.

In a plasmonic phototransistor fabricated by introducing source and drain electrodes, as well as patterned Au nonamer antennas, to the monolayer graphene sheet, Fang and co-workers demonstrated photoinduced n-doping of graphene by measuring the carrier density.^109^ The source–drain current *I* with respect to the backgate voltage *V*_g_ showed a minimum value when *V*_g_ was equal to Dirac voltage *V*_D_ due to charge neutrality if the graphene Fermi level is at the Dirac point. Under laser illumination, the *I*–*V*_g_ curves displayed a wavelength-dependent shift for the Dirac voltage *V*_D_ due to the plasmon-induced hot-electron transfer from the Au nonamer antennas to the graphene sheet (Figure 8a), leading to a prominent change of carrier density. The voltage shift correlates well with the absorption cross-section of the Au antennas (Figure 8b). Moreover, the doping effect can be controlled by varying the size of the plasmonic antenna and the laser power density.

Similarly, plasmon-induced hot-electron generation can be used to dope a MoS_2_ monolayer through the deposition of Au nanoparticles (Figure 8c).^110^ Under the resonant excitation of LSPR, both the absorption and photoluminescence spectra of the MoS_2_ monolayer exhibit a red-shift. The transfer of hot electrons from the Au nanoparticles to the MoS_2_ conduction band induced n-doping. The doping modulated the dielectric permittivity and increased the exciton binding energy of MoS_2_, which was then probed by the energy difference between the absorption and photoluminescence peak. The magnitude of spectral redshift, namely the doping density, increased monotonically with laser intensity (Figure 8d) and nanoparticle concentration.

#### 4.4.2 Structural Phase Transition

Interestingly, the resonant excitation of plasmon modes in Au nanoparticles deposited on an MoS_2_ monolayer can induce a transient reversible phase transition in MoS_2_ from the trigonal prismatic (2H) to the metallic octahedral (1T) (Figure 8e).^111^ The phase transition is revealed by the appearance of characteristic Raman modes of the octahedral phase, as well as a red-shift and intensity change to the photoluminescence (Figure 8f). The structural transition from the 2H to the 1T phase is induced by the destabilization of the lattice and the population of the Mo 4d orbitals with electrons originating from the deposited Au nanoparticles through hot-electron transfer.

## 5 Hot-Electron Transfer from a Plasmonic Metal to a Platinum-Group Metal

Platinum-group metals (PGMs), including Pt, Pd, Ir, Rh, and Ru, are catalytically more capable than plasmonic metals. However, PGMs tend to suffer from strong plasmon damping due to their large dielectric losses. Hence, the quality factor of their optical responses is low (weak plasmon band with low intensity and broad width). Integrating excellent plasmonic metals with PGMs can bring together high-quality LSPRs and catalytic-active surfaces. In general, PGMs such as Pt or Pd could be readily deposited on the hot spots of plasmonic nanocrystals through colloidal growth to achieve high efficiency for hot-electron injection. Alternatively, they can coat the entire surface of a plasmonic nanocrystal to maximize the number of active sites.

There are two ways for integrating plasmonic metals with PGMs: direct contact and noncontact. The direct contact leads to the formation of an Ohmic contact between the two materials, which is beneficial for hot-electron transfer across the interface. The direct contact can damp the LSPR and thereby affect the light-harvesting capability of the plasmonic nanocrystals. Another integration mode is to place PGMs near the plasmonic nanocrystals with a small gap (or separated by a dielectric spacer). The near-field coupling between these two units could enhance the light absorption of PGMs at the LSPR wavelength and drastically increase creation of hot electrons inside the PGMs and thus accelerate the kinetics of various catalytic reactions. The noncontact mode will not influence the LSPR of the plasmonic nanocrystals. In the contact mode, the near-field coupling effect always exists and also contributes to the hot-electron transfer.

### 5.1 Plasmonic Metal in Direct Contact with Platinum-Group Metal

**Figure 9:**
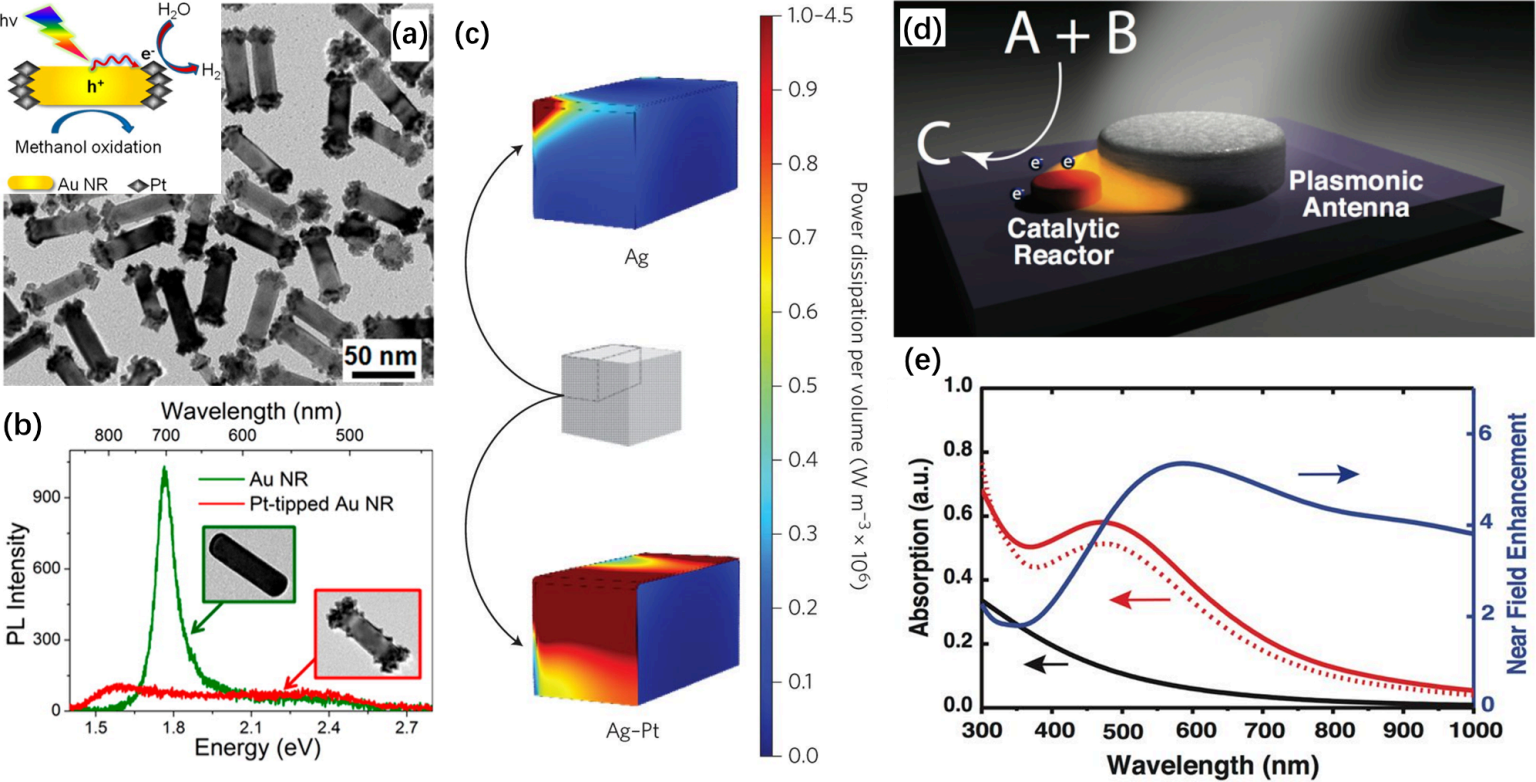
(a) Schematic and TEM image of Pt-tipped Au nanorods for photocatalytic H_2_ generation. The spatial separation of reduction and oxidation sites on the Pt-tipped Au nanorod results in an efficient charge separation and improved H_2_ production. (b) Single-particle photoluminescence quenching arising from hot-electron transfer from the excited Au nanorod to Pt. Reproduced with permission from ref (113). Copyright 2014 American Chemical Society. (c) Heat maps of power dissipation per volume at the LSPR peak of 455 nm for a Ag nanocube and 460 nm for the Ag@Pt nanocube. The result indicates that more energy is dissipated through absorption into the Pt shell compared with the case involving pure Ag. Reproduced with permission from ref (116). Copyright 2017 Springer Nature. (d) Schematic of an antenna–reactor complex comprising of a plasmonic antenna and a catalytic reactor in near-field coupling. (e) Absorption enhancement by the Pd unit in an Al–Pd antenna–reactor structure. The red solid curve shows the Pd absorption in the Al–Pd antenna–reactor complex calculated using finite-difference time-domain (FDTD) method, which exhibits an enhanced absorption around 500 nm relative to the isolated Pd (black solid curve). The red dashed curve is the isolated Pd absorption multiplied by electric field enhancement of Al nanoantenna (blue solid curve), which closely matches the red solid curve. Reproduced with permission from ref (117). Copyright 2016 Proceedings of the National Academy of Sciences USA.

Hot electrons can be extracted by a PGM in direct contact with the plasmonic metal to facilitate a chemical reaction catalyzed by the PGM. To this end, Wang and co-workers reported the use of Au–Pd bimetallic nanostructures to harvest visible and NIR light for the acceleration of a chemical reaction.^112^ The bimetallic system was consisted of Pd nanoparticles epitaxially attached to Au nanorods, mainly at the two ends. The intimate integration of plasmonic Au nanorods with catalytic Pd nanoparticles could accelerate Suzuki coupling reactions through efficient light harvesting by LSPR and then enrichment of hot electrons on the Pd surface. The spatial overlap between the plasmonic hot spots and catalytic sites, as well as the efficient transfer of hot electrons from Au to Pd, played an important role in achieving a high enhancement factor. Tachikawa, Majima, and co-workers also reported the controlled overgrowth of Pt on Au nanorods for the synthesis of Pt-tipped (tip-covered) and Pt-covered (fully covered) Au nanorods by altering the surfactant coating on the side surface (Figure 9a).^113^ The Pt-tipped Au nanorods exhibited much higher photocatalytic activity toward hydrogen generation compared to the Pt-covered samples under visible and NIR irradiations as a result of the strong longitudinal LSPR with hot spots located at two ends of the Au nanorod and effective transfer of hot electrons from Au to Pt surface. They further combined single-particle spectroscopy with photoluminescence quenching to verify the energy relaxation of the plasmon-induced hot electrons (Figures 9b). Other similar hybrid nanostructures, including the Pd-modified Au nanorods and Pt-modified Au nanoplates, have also been synthesized and utilized for plasmon-enhanced photocatalysis *via* hot-electron transfer.^114,115^

Linic and co-workers reported a comprehensive study of the photocatalytic activity of Ag@Pt core–shell nanocubes in the preferential oxidation of CO with excess H_2_.^116^ While Ag is known as the best-performing plasmonic metal, it tends to suffer from poor stability due to its vulnerability to sulfurization and oxidative etching. By conformally coating the surface of 75 nm Ag nanocubes with an ultrathin Pt shell of six atomic layers (or 1.2 nm thick), one can protect Ag from destruction by sulfurization or oxidative etching while presenting a viable catalytic surface. Both spectroscopic characteristics and theoretical simulations indicated that the contribution of absorption to the extinction of light by the Ag@Pt nanocubes was enhanced relative to the pristine Ag nanocubes. The energy of the visible light harvested by the plasmonic Ag cores could be selectively channeled into a catalytic reaction through an effective plasmon decay for the generation of hot electrons in the Pt shell due to its strong plasmon damping (Figure 9c).

### 5.2 Plasmonic Metal not in Contact with Platinum-Group Metal

Due to the field enhancement associated with LSPR excitation, the PGM next to a plasmonic nanocrystal will experience an enhanced excitation field and thus augmented absorption. The near-field coupling effect always exists regardless of direct contact or not between the PGM and the plasmonic metal. When separated from the plasmonic metal by air or a dielectric spacer, PGM can also experience the effect of hot electrons through near-field coupling.^117−120^ Even in the case with direct contact, the near-field coupling also contributes to the creation of hot electrons inside the PGM nanocrystals although there is no direct transfer of hot electrons. The advantage of noncontact mode is that the LSPR of the plasmonic nanocrystals would not be strongly damped by the PGM, which is beneficial to more effective light harvesting.

Nordlander, Halas, and co-workers demonstrated this concept through the fabrication of a Al–Pd antenna–reactor structure to enhance photocatalytic hydrogen dissociation (Figure 9d).^117,118^ Within such an antenna–reactor structure consisting of Pd-decorated Al nanocrystals (with 2- to 4 nm Al_2_O_3_ as a spacer), the near-field coupling between a plasmonic Al antenna (with an ultrathin oxide layer) and a catalytic Pd reactor led to enhanced light absorption in the Pd due to a “forced plasmon” effect and thus light-induced generation of hot electrons in the Pd component (Figure 9e). The photocatalytic hydrogen desorption shows a spectral response matching the antenna-induced local absorption cross-section of Pd, together with a superlinear power dependence.^117^ Moreover, the photocatalytic activity of the Al–Pd antenna–reactor complex exhibited selectivity toward acetylene reduction by hydrogen, and this selectivity was dependent on the irradiation power. With the increase in laser power density, the product of ethylene was greatly increased and the ethylene/ethane product ratio increased from ∼7 to ∼37. Significantly, the “reactor” can be readily configured to include different PGMs and their alloys, as well as many other metals, semiconductors, and even insulators, for the presentation of tunable surface chemistry and photocatalytic activity.

## 6 Hot-Electron Generation and Transfer Involving Other Plasmonic Materials

Plasmonic metals offer numerous advantages, including superior quality, chemical stability, and ease of surface modification. However, they are also characterized by higher costs and significant optical losses. Besides plasmonic metals,^121−124^ nonmetals, including nonstoichiometric copper chalcogenides (e.g., Cu_2–*x*_S),^125,126^ extrinsically doped metal oxides (e.g., Sn-doped In_2_O_3_),^127,128^ and oxygen-deficient metal oxides (e.g., WO_3–*x*_),^129,130^ also show tunable plasmonic properties. Compared to the plasmonic noble metals with a high and fixed free electron concentration (*ca*. 10^23^ cm^–3^), heavily doped semiconductors have varied carrier densities from 10^16^ to 10^21^ cm^–3^,^122^ and usually support LSPRs in the near-infrared region. They hold great promise for biomedical imaging and photothermal therapy due to the significantly deeper penetration of near-infrared light into biological tissues. Meanwhile, the carrier concentration of heavily doped semiconductors can be conveniently tuned by chemical doping, external electric/optical field and temperature to suit many scenarios involving modulation. Here we briefly discuss hot-carrier transfer in plasmonic hybrids comprised of p-type, nonstoichiometric copper chalcogenides and n-type, oxygen-deficient metal oxides, respectively.

**Figure 10:**
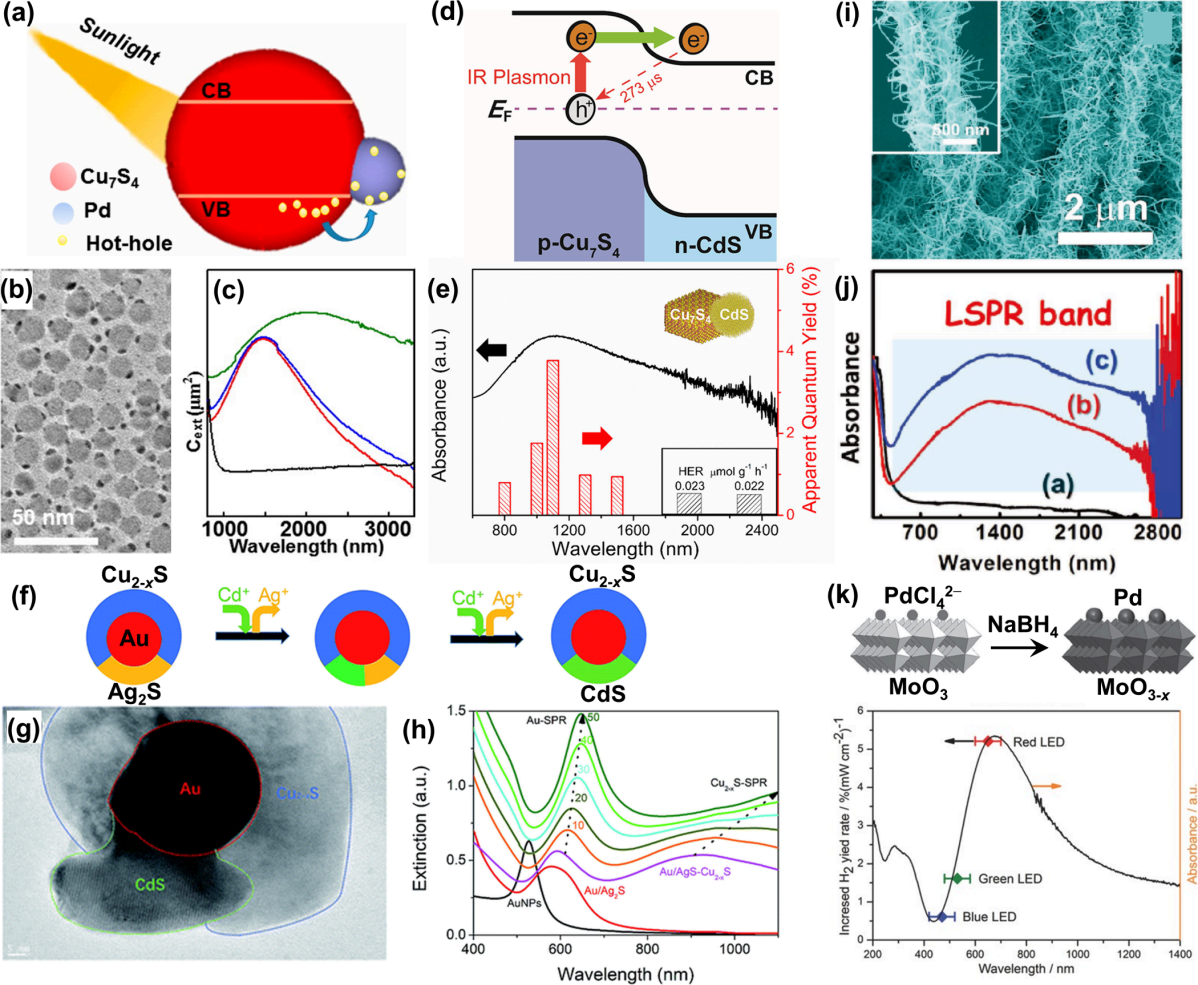
Hot-electron and hot-hole transfer involving nonmetal plasmonic nanomaterials. (a) Schematic of hot-hole transfer from Cu_7_S_4_ to Pd in Cu_7_S_4_@Pd nanoparticles. (b) TEM image of Cu_7_S_4_@Pd nanoparticles. (c) Extinction spectra of Cu_7_S_4_@Pd (green), physical mixture of Cu_7_S_4_ and Pd (blue), Cu_7_S_4_ (red), and Pd (black). Reproduced with permission from ref (131). Copyright 2015 American Chemical Society. (d) Hot-electron injection at the p–n heterojunction of CdS-Cu_7_S_4_ nanoparticles upon plasmon excitation by NIR light. (e) Absorption spectrum and apparent quantum yield (AQY) for the photocatalytic H_2_ evolution reaction on the CdS-Cu_7_S_4_ under the illumination of a monochromic light (6 mW·cm^–2^). Reproduced with permission from ref (132). Copyright 2019 American Chemical Society. (f) Preparation of Au@CdS-Cu_2–*x*_S core–shell nanoparticles through a cation exchange reaction. (g) TEM images of Au@CdS-Cu_2–*x*_S core–shell nanoparticles. (h) Extinction spectra of Au@CdS-Cu_2–*x*_S prepared with different amounts of CdCl_2_. Reproduced with permission from ref (133). Copyright 2019 Royal Society of Chemistry. (i) SEM image of W_18_O_49_–TiO_2_ branched heterostructures for enhancing the catalytic generation of H_2_ from NH_3_BH_3_. (j) Absorption spectra of TiO_2_ (black), W_18_O_49_–TiO_2_ (red), and W_18_O_49_ (blue), respectively. Reproduced with permission from ref (134). Copyright 2018 Wiley-VCH. (k) Pd-MoO_3–*x*_ hybrid structures prepared through the reduction of PdCl_4_^2–^ by NaBH_4_ in the presence of MoO_3_. Absorption spectrum of Pd-MoO_3–*x*_ hybrid structures and the increased H_2_ yield rate relative to dark condition by LED irradiation at three different wavelengths. Reproduced with permission from ref (135). Copyright 2015 Wiley-VCH.

Self-doped binary copper chalcogenides (e.g., Cu_2–*x*_S, Cu_2–*x*_Se, and Cu_2–*x*_Te) contain Cu vacancies, creating holes in the top of the valence band and thus supporting LSPR in the NIR region through the collective oscillation of holes. Their coupling to PGMs and semiconductors can result in a plasmonic enhancement effect due to hot-carrier transfer. Wang, Huang, and co-workers reported that the strong NIR plasmonic absorption of Cu_7_S_4_@Pd facilitated hot-hole transfer from Cu_7_S_4_ to Pd, which subsequently promoted the catalytic reaction on Pd surface (Figure 10a).^131^ The Cu_7_S_4_@Pd hybrids were fabricated by attaching 4.3 nm Pd nanoparticles to 14 nm Cu_7_S_4_ nanoparticles, red-shifting the absorption peak to around 2000 nm (Figure 10, b and c). Under solar illumination with a power density as low as 40 mW/cm^2^, nearly 80–100% conversion was achieved within 2 h for three types of photocatalytic organic reactions, including Suzuki coupling, hydrogenation of nitrobenzene, and oxidation of benzyl alcohol. Sakamoto, Teranishi, and co-workers constructed a p–n heterojunction from Cu_7_S_4_ and CdS nanocrystals to create an electric field capable of promoting charge separation.^132^ In the photocatalytic hydrogen generation reaction, the hot electrons generated in the plasmonic Cu_7_S_4_ nanocrystals at an LSPR peak of 1115 nm were injected into the conduction band of the CdS unit to react with H_2_O for the generation of H_2_ while the holes were consumed by the sacrificial agents (Figure 10d). The apparent quantum yield reached 3.8% at 1100 nm, and spectroscopic analysis revealed that the plasmon-induced hot-electron injection at the p–n heterojunction led to exceptionally long-lived charge separation (>273 μs, Figure 10e). In another study, a lateral p–n heterojunction comprised of CdS and Cu_2–*x*_S was successfully grown on Au nanoparticles through a cation exchange reaction (Figure 10f).^133^ The Au@CdS–Cu_2–*x*_S nanoparticles, with two LSPR peaks located in the visible (for Au) and NIR (for Cu_2–*x*_S) regions nicely integrates two plasmonic materials (Au and Cu_2–*x*_S), two semiconductor materials (CdS and Cu_2–*x*_S), and three interfaces (Au–CdS, Au–Cu_2–*x*_S, and CdS–Cu_2–*x*_S) into one heteronanostructure (Figure 10, g and h). Compared to the conventional double-shelled particles, the new design featuring a lateral heterojunction in the shell is advantageous in that both the semiconductors are directly in contact with the reactants during the photocatalytic reaction, and the separated electrons and holes can participate in the reduction and oxidation reactions, respectively.

Different from the copper chalcogenides, self-doping in the oxygen-deficient metal oxides produces electrons in the conduction band and thereby n-doping. Dong and co-workers fabricated plasmonic W_18_O_49_ nanowires as branches on TiO_2_ electrospun nanofibers (serving as the backbone) through a solvothermal method (Figure 10i).^134^ The W_18_O_49_ nanowires, with a blue color, exhibited a dual-absorption feature, including a bandgap absorption below 400 nm and an intense plasmonic absorption across the whole NIR range (Figure 10j). Upon LSPR excitation with low-energy NIR photons, the W_18_O_49_–TiO_2_ branched heteronanostructures exhibited enhanced photocatalytic activity toward H_2_ generation from NH_3_BH_3_. On account of an ultrafast transfer of hot electrons from the W_18_O_49_ branches to the TiO_2_ backbones, the reaction occurred within a time frame on the order of 200 fs. Yamashita and co-workers presented a catalytic–plasmonic hybrid comprised of Pd-MoO_3–*x*_, with Pd nanoparticles anchored to MoO_3–*x*_ plates, by reducing a mixture of MoO_3-x_ plates and PdCl_4_^2–^ ions through an impregnation process involving H_2_PdCl_4_ (Figure 10k).^135^ The Pd-MoO_3–*x*_ hybrids, with an intense LSPR in the visible region near 640 nm, exhibited plasmon-enhanced catalysis toward NH_3_BH_3_ hydrolysis and Suzuki–Miyaura coupling reactions under visible light. The enhancement was attributed to the creation of hot electrons in the MoO_3–*x*_ unit and their subsequent injection into the adjacent Pd nanoparticles. Wang and co-workers demonstrated a Schottky-barrier-free plasmonic semiconductor based on MoO_3–x_ spheres with rich oxygen vacancies (OVs) for N_2_ photofixation.^136^ The OVs in the MoO_3–x_ spheres not only serve as active sites for the chemisorption and activation of N_2_ molecules but also contribute to an increased free charge carrier density. This leads to the occurrence of LSPR, generating hot charge carriers to drive the reduction of N_2_ to NH_3_. The apparent quantum efficiency (AQE) exceeds 1% between 600–1064 nm, with the highest value of 1.24% recorded at 808 nm. The absence of a Schottky barrier in this plasmonic semiconductor enables the free transportation of hot charge carriers, while the defect states created by the OVs effectively capture hot electrons and thereby prevent their recombination with holes.

## 7 Synthesis of Plasmonic–Catalytic Hybrid Nanostructures

From the above discussion, it is not difficult to understand why there is an urgent need to construct plasmonic–catalytic hybrid systems with effective hot-electron transfer for photocatalysis and related applications. Specifically, the plasmonic–catalytic hybrid systems based on bimetallic and metal–semiconductor nanostructures stand out as two unique platforms for bringing together an effective light-harvesting antenna and a catalytically active surface. In this section, we briefly discuss how to rationally fabricate these two hybrid systems with the desired features or properties using wet-chemical methods. Readers should consult major review articles for detailed discussions on these synthetic methods.

In general, the synthesis of such a hybrid system involves the deposition of either a catalytic metal or its semiconductor counterpart on a plasmonic nanocrystal. Depending on the application, one should choose plasmonic components featuring a proper combination of composition, size, shape, morphology, and internal structure as both the production rate and energy distribution of hot electrons are strongly dependent on these parameters. Thanks to the efforts from many groups, plasmonic nanocrystals (including those made of Au, Ag, and Cu) can now be synthesized using wet-chemical methods to provide diverse shapes or morphologies, such as spheres, cubes, rods, wires, and thin plates, among others.^137−139^ Some of these shape-controlled nanocrystals can also be prepared with tunable sizes. With the implementation of various theoretical methods, the LSPR peak position, optical cross sections, local field enhancement, and spatial distribution of hot spots can all be calculated for the plasmonic nanocrystals of interest before any synthetic effort is attempted. For the deposition of the catalytic component, it can be initiated and confined to certain regions on the surface of a plasmonic nanocrystal by optimizing the experimental conditions. In many cases, the deposition can be selectively initiated from the plasmonic hot spots to ensure a strong local field enhancement and thus efficient hot electron injection. Alternatively, coating the entire surface of a plasmonic nanocrystal with a thin shell of the catalytic material will provide the largest active surface while shortening the distance for the energized electrons to move from the interface to the active sites. Thanks to recent progress in colloidal synthesis, plasmonic–catalytic hybrid nanostructures with a set of specified parameters such as composition, morphology, size, and symmetry can be designed and rationally synthesized. As such, there are multiple ways to achieve highly efficient hot-electron generation and transfer, as well as high conversion efficiency, to drive energy flow through the pathway of photon–electron–chemical energy. Here we briefly review some typical examples of hybrid nanostructures with controlled compositions, structures, and/or morphologies. The controlled synthesis of metal–metal and metal–semiconductor hybrids is discussed separately.

### 7.1 Metal–Metal Hybrids

Relative to their metal–semiconductor counterparts, controlled overgrowth of PGMs on plasmonic metal nanocrystals is much easier to implement because all of them except Ru share the same face-centered cubic (*fcc*) structure, together with relatively small lattice mismatching (<5%). Colloidal synthesis of nanocrystals with controlled sizes and shapes has been well developed for both the plasmonic (e.g., Au, Ag, and Cu) and catalytic (e.g., Pt, Pd, and Rh) metals. The tight controls in terms of size and shape have led to plasmonic nanocrystals with predictable LSPR properties for light harvesting and optimal distribution of hot spots for hot-electron generation. At the same time, the size and type of crystal facets exposed on the catalytic component can be leveraged to engineer the activity and/or selectivity toward various reactions. Through site-selective or morphologically controlled deposition of a catalytic metal on a plasmonic metal nanocrystal, one could achieve a highly efficient energy flow from the light-harvesting plasmonic core to the catalytic active sites *via* hot-electron transfer.

**Figure 11:**
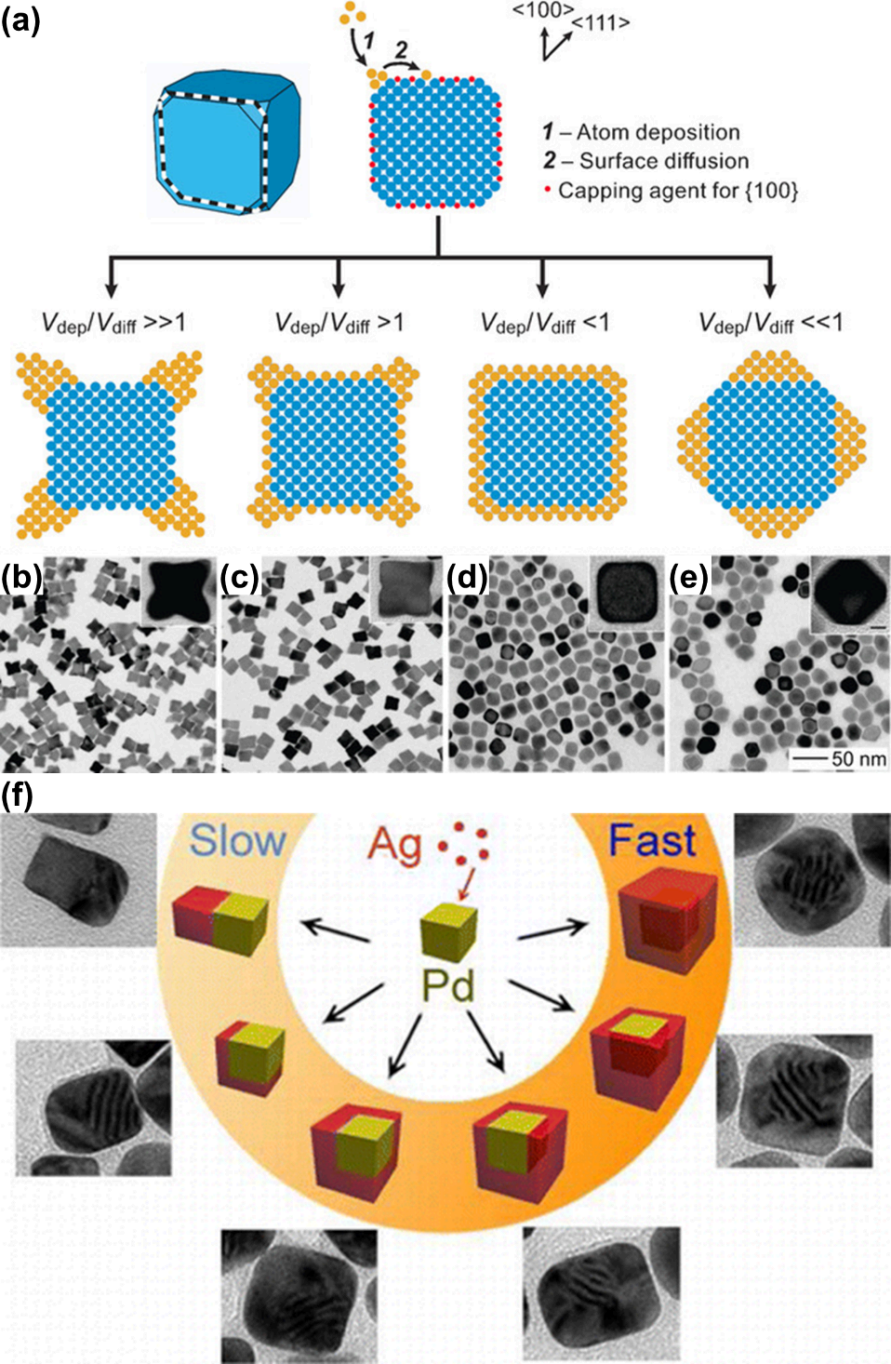
(a) Metal deposition on a cubic seed under four different kinetic conditions: *V*_dep_/*V*_diff_ ≫ 1, *V*_dep_/*V*_diff_ > 1, *V*_dep_/*V*_diff_ < 1, and *V*_dep_/*V*_diff_ ≪ 1. *V*_dep_ and *V*_diff_ represent the deposition rate and surface diffusion rate, respectively. (b-e) TEM images of four distinctive types of Pd nanocrystals synthesized at temperatures of b) 0, c) 22, d) 50, and e) 75 °C. Reproduced with permission from ref (143). Copyright 2017 Wiley-VCH. (f) Schematic illustration and TEM images showing Ag growth on different numbers of faces on a Pd cubic seed by carefully controlling reaction kinetics. Reproduced with permission from ref (144). Copyright 2012 American Chemical Society.

The deposition of a second metal on a metal nanocrystal is relevant to seed-mediated growth. The growth pattern and the morphology of the final product are dependent on reaction kinetics. First of all, homogeneous nucleation of the newly formed atoms should be suppressed to ensure heterogeneous nucleation only for the expected overgrowth, and this can be realized by slowing down the reduction rate through a combination of reducing the precursor addition rate, using a weaker reductant, and lowing the reaction temperature. The growth pattern is determined by the competition between conformal growth and site-selected growth, corresponding to the layer-by-layer or Frank-van der Merwe growth mode and island or Volmer–Weber growth mode (or a mixed Stranski–Krastanov growth mode), respectively.^140−143^ A typical example is shown in Figure 11a, where the interplay between the atomic deposition rate and the surface diffusion rate leads to different growth modes, including island deposition on high-energy sites, frame structures with high-index facets, conformal core@shell structures maintaining the initial shape, and morphology variations with different types of facets. The preferential sites for atom deposition are commonly the high-energy sites like vertices, edges, and twin boundaries, or the less-capped sites when the surface is passivated by a capping agent. In the case of Pd nanocubes, all the {100} side faces are passivated by Br^–^ ions so that atom deposition prefers to occur at the {111} vertices and {110} edges. The four morphologies shown in Figure 11, b-e can be realized by manipulating the surface diffusion rate through reaction temperature and/or tuning the atomic deposition rate through the precursor injection rate. Hybrid nanostructures involving both conformal growth and site-selected growth modes have been actively explored in photochemical applications. For the Ag@Pt core–shell nanocube, the ultrathin Pt shell formed *via* conformal growth on an Ag nanocube can protect the Ag core from oxidative etching while effectively generating hot electrons in the catalytic Pt shell for surface catalytic reactions.^116^ On the other hand, the Pt-tipped Au nanorod with site-selected deposition of Pt on both ends of the Au nanorod showed more efficient photocatalytic activity than its counterpart fully covered by Pt, owing to the large local field enhancement on the tip regions induced by the intense longitudinal LSPR, as well as the efficient charge separation along the longitudinal direction.^113^

The symmetry of the metal–metal hybrid is another interesting parameter for control.^144−150^ As shown in Figure 11f, the deposition of Ag on Pd nanocubes can be kinetically controlled to obtain concentric nanocubes, nonconcentric nanocubes, and dimers with a Janus configuration.^144,145^ The reaction kinetics can be experimentally manipulated by controlling the injection rate of the Ag(I) precursor, type of reducing agent, pH value, and reaction temperature. Under proper kinetics, the newly formed atoms selectively nucleated and then epitaxially grew from one side face (slowest reaction) to six side faces (fastest reaction) of a Pd nanocube. The concentric distribution is the same as the aforementioned conformal core–shell structure with symmetrical morphology, while the nonconcentric distribution is a result of symmetry breaking and asymmetrical growth. The asymmetrical growth is achieved by controlling the supply of newly formed atoms and it will be assisted by the lattice mismatch between the two metals. The asymmetrical products could expose both metals in photochemical reactions to construct a photosynthetic device with capabilities for both oxygen evolution and hydrogen evolution reactions,^87^ and the ratio between the exposed areas of the two metals can be manipulated to possibly favor different chemical reactions. Overall, the integration of plasmonic metals with catalytic metals in different and precisely controlled configurations offers many possibilities to enhance the photocatalytic performance.

### 7.2 Metal–Semiconductor Hybrids

**Figure 12:**
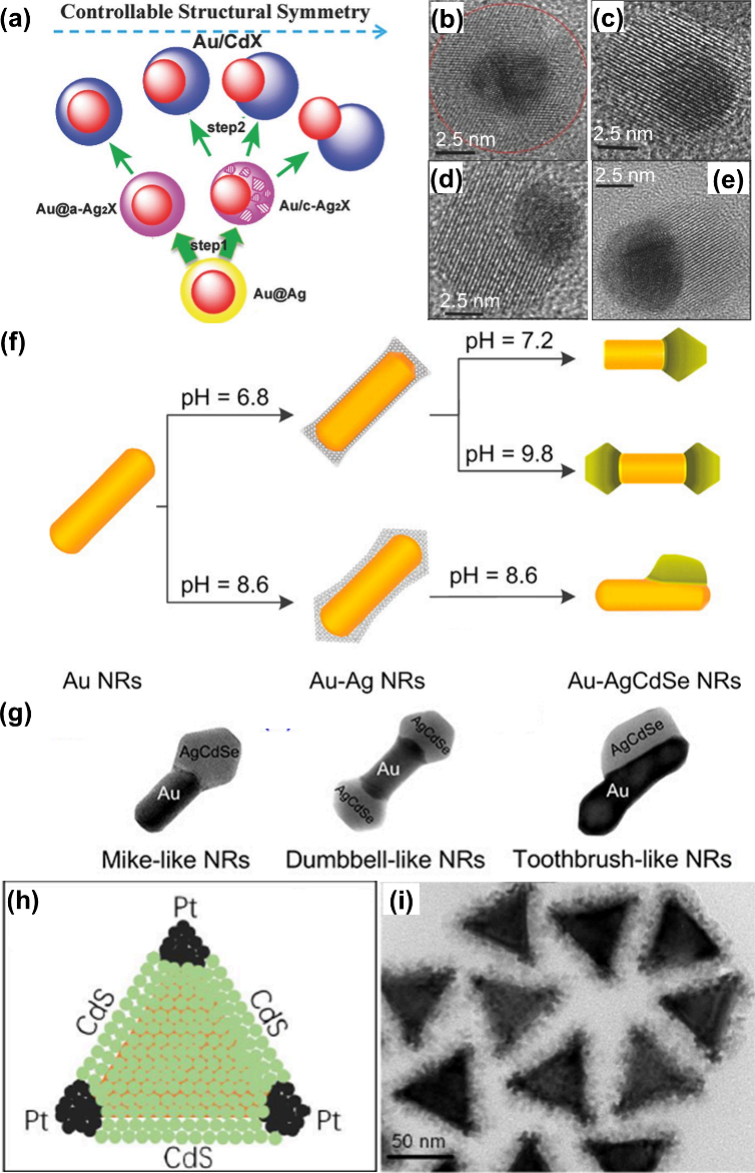
(a) Schematic illustration of controllable structural symmetry in Au/CdX (X = S, Se and Te) hybrids with large lattice mismatch by two steps of *in situ* chemical transformation. The a-Ag_2_X and c-Ag_2_X correspond to amorphous and crystalline Ag_2_X, respectively. (b-e) HRTEM images of the Au/CdS hybrids with controllable structural symmetry: (b) Concentric core–shell; (c) Nonconcentric core–shell; (d, e) Heterodimers. The scale bar is 2.5 nm. Reproduced with permission from ref (158). Copyright 2013 Wiley-VCH. (f) Schematic illustration of growing three different types of Au–AgCdSe hybrid nanorods by manipulating the pH value. (g) TEM images of mike-like, dumbbell-like, and toothbrush-like hybrid nanorods, respectively. Reproduced with permission from ref (159). Copyright 2012 American Chemical Society. (h) Schematic illustration and (i) TEM image of Au–Pt–CdS heteronanostructures. Reproduced with permission from ref (168). Copyright 2016 Wiley-VCH.

The lattice mismatch between metals and semiconductors is a big obstacle for fabricating metal–semiconductor hybrids; many strategies have been used to overcome this obstacle. Two effective approaches have been reported to construct metal–semiconductor heteronanostructures with well-defined morphology: *i*) using an intermediate metal or semiconductor layer to assist the growth of a semiconductor on a plasmonic metal;^151−153^ and *ii*) using molecule–cation complexes to transport precursor ions to the surface of metal nanocrystals for facile semiconductor deposition.^154−156^ Symmetrical core–shell nanoparticles are usually the products of these approaches. Asymmetrical morphology is a challenging and yet attractive goal.^157−162^ When the intermediate-layer method is used, the symmetry of the metal–semiconductor hybrid can be controlled by adjusting the intermediate layer and/or the reaction rate of the growth process. For instance, the structural symmetry of Au/CdX (X = S, Se, and Te) hybrid heteronanostructures could be tuned from symmetric core–shell to asymmetric heterodimer using a nonepitaxial synthetic route (Figure 12a).^158^ This synthesis started with a concentric Au@Ag core–shell nanoparticle, followed by *in situ* conversion of the Ag shell to Ag_2_X and a cation-exchange process to further transform Ag_2_X into monocrystalline CdX.^151^ The crystallinity (and morphology) of Ag_2_X could be controlled from amorphous (concentric) to partially crystalline (nonconcentric) by adjusting the sulfur precursor and increasing the reaction temperature, leading to different lattice mismatches between the Au core and the Ag_2_X shell. The concentric and amorphous Ag_2_X shell resulted in the formation of a concentric Au@CdX core–shell nanoparticle (Figure 12b). However, the polycrystalline Ag_2_X induced a larger phase separation between Au and CdX for the purpose of reducing the interfacial and grain boundary energies, giving rise to the formation of asymmetric heterodimers (Figure 12, c-e). The tuning of structural symmetry has also been demonstrated in 1D Au–AgCdSe nanorods, as shown in Figure 12f.^159^ In this case, an intermediate Ag layer was first deposited on a Au nanorod. The Ag layer was subsequently selenized and the resultant Ag_2_Se served as a substrate for the overgrowth of CdSe. The selenide nanocrystals could be site-selectively grown on one end, two ends, and side surface of the Au nanorod by controlling the pH value during the deposition of the intermediate Ag layer and the growth of selenide. The I- and shuttle-shaped Au–Ag nanorods were obtained when the pH was tuned to 6.8 and 8.6, respectively. The growth pattern of Ag determined the sites for the formation of selenides. Based on the I-shaped Au–Ag nanorods, asymmetric mike-like nanorods were obtained when the growth rate, controlled by the pH value, was slowed down, while symmetric dumbbell-like nanorods formed when the growth rate was adjusted to fast. (Figure 12g). The overgrowth of AgCdSe on the shuttle-shaped Au–Ag nanorods led to the formation of asymmetric toothbrush-like nanorods (Figure 12g).

Furthermore, multicomponent hybrids with synergetic effect could be realized using the site-selected growth strategy.^163−171^ For example, in a tricomponent Au–Pt–CdS hybrid (Figure 12, h and (i),^168^ Pt nanoparticles could be selectively grown on the three tips of Au nanotriangles because the tip regions were covered by surfactant molecules at a lower coverage density. Afterward, CdS layers were deposited on the still exposed regions. In these Au–Pt–CdS heteronanostructures, plasmonic metal (Au), catalytic metal (Pt), and active semiconductor (CdS) are integrated together. Meanwhile, three types of heterointerfaces (Au–Pt, Au–CdS, and CdS–Pt) are created. The hot-electron generation and multipathway electron transfer could greatly enhance the performance of photochemical applications. It should be noted that the quality and crystallinity of the interfaces are significantly important for the interfacial charge transfer, while the synthesis of high-quality interfaces is still challenging because of the typical large lattice mismatch between metals and semiconductors.^172,173^

## 8 Conclusions and Outlook

In this review, we briefly discuss the fundamentals and applications related to the generation, transfer, and utilization of hot electrons. Specifically, we highlight recent progress in harvesting the plasmon-induced hot electrons for various applications, with a focus on the transfer of such energetic electrons from plasmonic nanocrystals directly to organic molecules for photochemical reactions, as well as to semiconductors and metals acting as catalytic substrates. Using many attractive designs of plasmon-involved hybrid nanomaterials, the underlying physical mechanisms of indirect and direct transfer of hot electrons are introduced, together with interesting observations related to absorption, photoluminescence, photoresponsivity, and transient absorption kinetics. Finally, we briefly touch on wet-chemical methods for both conformal and site-selected growth to generate colloidal hybrid nanostructures with controlled morphology and symmetry.

Despite the remarkable progress, challenges and opportunities still remain for this relatively new field of research, especially in the context of rational synthesis, device design, and application. In terms of synthesis, experimental controls over the plasmonic components made of Au, Ag, and Cu have been fully developed, with their spectral responses covering both visible and NIR regions. In the UV region, Al is a promising candidate albeit it is still challenging to synthesize Al nanocrystals with controlled shapes and thus desired LSPR properties.^39,174^ On the other hand, the synthesis of plasmonic nanocrystals from materials rather than metals remains to be fully explored because of their weaker plasmonic response relative to metals. Despite the progress in controlling the synthesis of plasmonic nanocrystals, there still exist challenges when applying the synthetic protocol to a hybrid system. For the deposition of a catalytic component onto plasmonic nanocrystals, Au has been the most commonly used material for its extremely high stability against oxidation. In comparison, it would be more challenging to apply both Ag and Cu to the deposition of another metal or a semiconductor *via* seed-mediated growth due to their susceptibility to oxidation or sulfuration. At the current stage of development, only a limited set of catalytic metals or semiconductors can be deposited with controlled dimensions, morphology, and symmetry. There are many opportunities in extending the kinetic control to other combinations of materials, especially with regard to the metal–semiconductor hybrids.

In general, a carefully designed device has to be fabricated using hybrid nanostructures in order to achieve efficient utilization of the energetic carriers. Despite numerous reports on hot electrons, studies of the collection and utilization of hot holes are few. Hole transport is slow, and the transport distance of hot holes is shorter than that of hot electrons. The hot holes left behind in the plasmonic nanocrystal after hot-electron transfer are usually scavenged by sacrificial reagents. The scavenge of hot holes plays an important role in balancing charges and suppressing oxidative corrosion of the plasmonic nanocrystal. The efficiency of a plasmonic device can be augmented by rationally optimizing the configuration of a plasmonic hybrid to expedite the hot-hole kinetics simultaneously. It is interesting to note that the direct hot-electron transfer in a strongly coupled plasmon–exciton nanosystem has a higher efficiency than the indirect process. In this case, the coherent coupling induces highly efficient energy transfer and swap in the hybridized states. This result suggests a new direction for further exploration of the plasmon-mediated energy conversion and collection of the energetic carriers.

The unique capability of plasmonic light-harvesting implies a great potential for hot-electron collection. However, the ultrafast nature of plasmon decay and hot-electron relaxation indicates that there are many obstacles to optimizing applications based on hot electrons. Meanwhile, the mechanism underlying a plasmon-enhanced process is very complicated because the hot-electron phenomenon is often mixed with other plasmonic effects such as local field enhancement, light-trapping of plasmonic scattering, PRET, and photothermal heating. For a better understanding of the nuances of hot-electron transfer, one can rely on time-resolved transient spectroscopy and spatially resolved functional imaging to detect plasmon decay and hot-electron relaxation. As previously reported in the literature, femtosecond transient spectroscopy covering the visible and middle-infrared regions, combined with excitation polarization dependence, was utilized *via* a pump–probe scanning technique to elucidate the mechanism of direct hot-electron transfer through the PICTT pathway.^98,99^ Additionally, single-particle imaging techniques based upon photoluminescence and dark-field scattering were deployed to examine the transfer of hot electrons between plasmonic metals and PGMs.^113−115^ To this end, surface-enhanced Raman scattering (SERS) is a sensitive method capable of identifying molecular fingerprints for *in situ* monitoring of plasmon-mediated chemical reactions because a plasmonic nanostructure is also a good substrate for SERS. The adoption of *in situ* SERS spectroscopy enables the real-time and label-free detection of reaction products^52^ while offering insights and evidence into the distinctive mechanism of hot-electron excitation and the selective activation of specific chemical bonds within plasmon-molecule coupling complexes.^59^ At other fronts, the plasmon-induced hot electrons have been explored to drive a chemical reduction for the initiation of nanocrystal growth.^175,176^ Interestingly, an electrically driven plasmonic nanorod array has been demonstrated to control chemical reactions in the nanoscale gaps using tunnelling-driven generation of hot electrons.^177^ This interesting observation suggests a new promise for extending the applications of plasmon-induced hot electrons to a greater scope beyond photodetection, photovoltaics, and photocatalysis.
